# Towards an experimental classification system for membrane active peptides

**DOI:** 10.1038/s41598-018-19566-w

**Published:** 2018-01-19

**Authors:** G. D. Brand, M. H. S. Ramada, T. C. Genaro-Mattos, C. Bloch

**Affiliations:** 1 Laboratório de Espectrometria de Massa, Embrapa Recursos Genéticos e Biotecnologia, Brasília, DF Brazil; 2 Laboratório de Síntese e Análise de Biomoléculas, Instituto de Química, Universidade de Brasília, Brasília, DF Brazil; 3 Departamento de Biologia Celular, Instituto de Ciências Biológicas, Universidade de Brasília, Brasília, DF Brazil; 4 Pós-Graduação em Ciências Genômicas e Biotecnologia, Universidade Católica de Brasília, Brasília, DF Brazil; 5 Chemistry Department, Vanderbilt University, Nashville, TN USA

## Abstract

Mature proteins can act as potential sources of encrypted bioactive peptides that, once released from their parent proteins, might interact with diverse biomolecular targets. In recent work we introduced a systematic methodology to uncover encrypted intragenic antimicrobial peptides (IAPs) within large protein sequence libraries. Given that such peptides may interact with membranes in different ways, resulting in distinct observable outcomes, it is desirable to develop a predictive methodology to categorize membrane active peptides and establish a link to their physicochemical properties. Building upon previous work, we explored the interaction of a range of IAPs with model membranes probed by differential scanning calorimetry (DSC) and circular dichroism (CD) techniques. The biophysical data were submitted to multivariate statistical methods and resulting peptide clusters were correlated to peptide structure and to their antimicrobial activity. A re-evaluation of the physicochemical properties of the peptides was conducted based on peptide cluster memberships. Our data indicate that membranolytic peptides produce characteristic thermal transition (DSC) profiles in model vesicles and that this can be used to categorize novel molecules with unknown biological activity. Incremental expansion of the model presented here might result in a unified experimental framework for the prediction of novel classes of membrane active peptides.

## Introduction

Genomes encode many proteins with borderline aqueous solubility that may partition into hydrophobic media or phospholipid membranes as soon as they are encountered^1^. Correspondingly, proteins have segments that are themselves capable of membrane adsorption, either as integral transmembrane components or, following enzymic digestion from parent proteins, as peptide fragments with propensity for various additional activities such as nucleation of amyloid plaques by preamyloid toxins (PATs), membrane translocation by cell-penetrating peptides (CPPs) or even membrane disruption by antimicrobial peptides (AMPs)^2–5^. Despite significant sequence and structure differences, these membrane active peptides share similarities regarding some physicochemical properties^2,6^. Recently, our group developed a methodology to uncover fragments of proteins that, once released from their original protein scaffold, can exert antimicrobial activity by plasma membrane disruption, in ways similar to antimicrobial peptides from conventional biological sources^4^. Indeed, such fragments derived from proteolysis, designated Intragenic Antimicrobial Peptides (IAPs), are currently being explored in biotechnological processes related to agriculture, human health and food conservation. Our methodology relies in two complementary steps: (1) the filtering of genomes/protein collections using a bioinformatic tool, “Kamal”^4,7^, which searches protein databases for fragments using a predefined set of physicochemical properties; and (2) an experimental classification tool to identify sets of IAPs which induce comparable disturbances in model phospholipid membranes. The latter was conceived to gain further insight in peptide-membrane interactions and to serve as a feedback to the selection process by refining the physicochemical parameters associated with a particular biological activity. The classification of putative antimicrobial peptides is based on differential scanning calorimetry (DSC), which is a powerful non-perturbing technique for the study of protein/peptide interactions with biological membranes^8–10^.

It is long known that phospholipid membranes react to adsorbent molecules with respect to their physicochemical nature^8^. Actually, inorganic ions, alkanols, fatty acids, organic acids, and detergents produce class-specific changes in membrane thermal profiles^8^. These disturbances are related to the position of the additive along the bilayer: hydrophilic molecules are adsorbed preferentially in the phospholipid head group region, while more hydrophobic molecules disturb the inner hydrocarbon core of membranes^4,8,9^. Membrane active peptides bind membranes in different modes, mechanisms and with variable consequences^9^. It is believed that there are at least 3 different permeation mechanisms only for antimicrobial peptides, with variable binding orientation, depth of penetration, promotion of membrane thinning, and peptide structuration, etc.^11^. The uncovering of similar peptide-induced membrane thermal profiles associated with a biological activity, such as antimicrobial, can be used for the classification of novel putative membrane active molecules^4^. Moreover, following the same rationale, similar effects on the main phase transition of membranes also indicate similarities in the physicochemical properties that are relevant for membrane interaction.

Here we report on the validation and expansion of DSC as a classification tool for membrane active peptides. In a previous study, we used DSC to group fifteen putative IAPs and eleven AMPs according to similarities in peptide-induced thermal profiles of model membranes^4^. Peptides from one group, composed of known AMPs, perturbed the hydrocarbon core of model membranes, underwent conformational change to more α-helical segments upon membrane association, and presented high antimicrobial activity^4^. The physicochemical properties of this particular group, namely, average hydrophobicity, hydrophobic moment, net charge, among others, were fed back to the Kamal filtering algorithm^4^, allowing us to identify a further set of novel IAPs from plant genomes that serve as a basis for the current work^7^. Please refer to the literature for further details on the Kamal software and the physicochemical criteria used for the uncovering of IAPs^4,7^. The biological activities of these novel peptides are described in an accompanying report^7^, whilst here we focus on their physicochemical properties. Future expansion of the currently presented methodology will assist the discovery of protein fragments with cell-penetrating and preamyloid activities and may provide a unified experimental framework for membrane active molecules.

## Results

Twenty-one novel putative IAPs were filtered from the genomes of *Theobroma cacao, Gossypium raimondii*, *Citrus sinensis, Arabidopsis thaliana* and *Zea mays*, and selected for solid phase synthesis and testing, as reported in detail in the accompanying paper^7^. Many of these peptides were found to be as active as AMPs from conventional sources. These IAPs, along with six known AMPs (DS01^12^, Mag-2a^13^, Nattererin-1^14^, Ascaphin-08^15^, PS-2^16^, HSP-04^4^), were incubated separately with Large Unilamellar Vesicules (LUVs) comprising either dimyristoylphosphatidylcholine (DMPC) alone or 2:1 dimyristoylphosphatidylcholine:dimyristoylphosphatidylglycerol (DMPC:DMPG) mixtures, and the resulting peptide-phospholipid LUVs were evaluated by DSC and circular dichroism (CD). DSC data were submitted to a principal component analysis (PCA) followed by hierarchical clustering analysis (HCA), revealing three peptide clusters. Two clusters presented significant antimicrobial activity versus Gram-positive and -negative bacteria, and yeasts. A meta-analysis encompassing the effect of the 27 current and the 25 previously evaluated peptides on LUVs thermal transitions was conducted, with detailed outcomes as follows.

### Novel IAPs and AMPs induce similar alterations in the main phase transition of model phospholipid membranes

**Figure 1:**
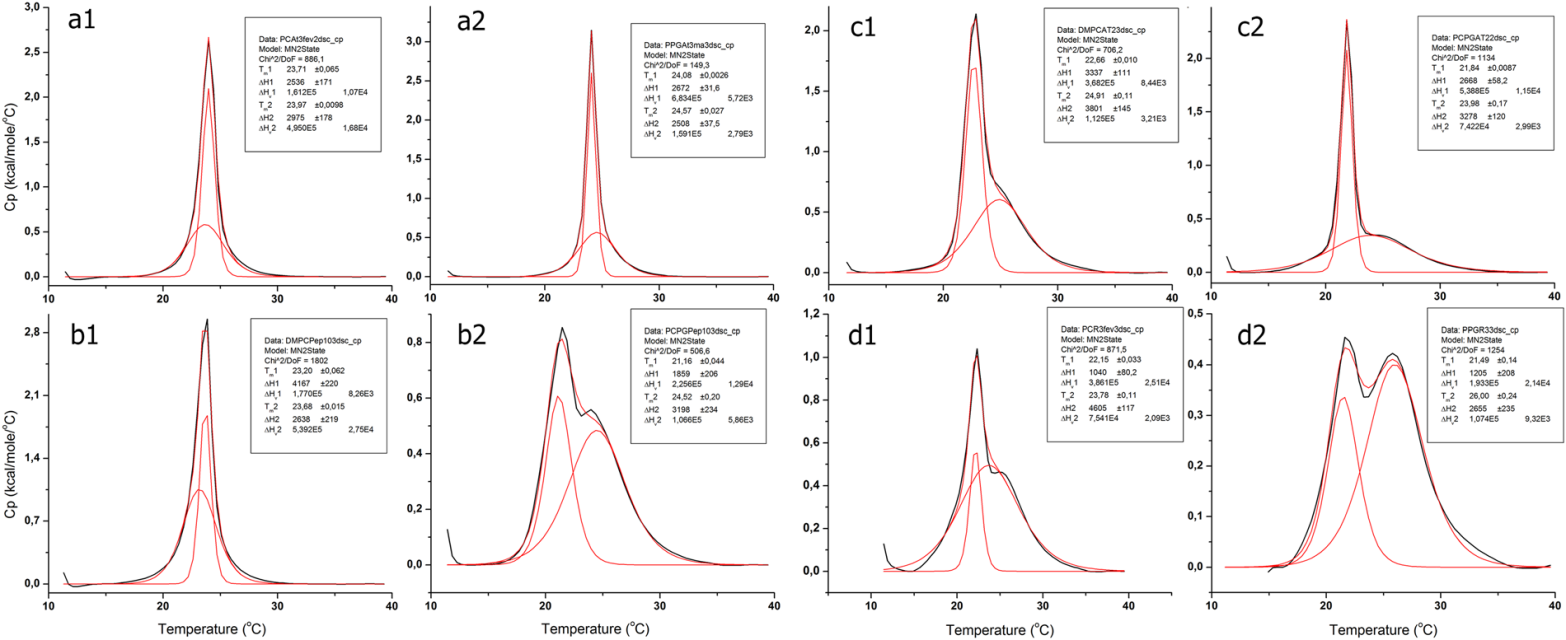
IAPs disturb the main phase transition of DMPC and 2∶1 DMPC:DMPG model membranes. DSC scans of phospholipid LUVs mixed with 4 mol% peptides. Thermograms were normalized to the lipid mass and the P′_β_→L_α_ transition was fit to a non-two state transition with two peaks. The experimental data are represented as a black line and model fitting is in red. Thermograms for the peptide At03 added to DMPC and 2:1 DMPC:DMPG LUVs are shown in insets (**a1** and **a2**), respectively. Thermograms depicting the effect of peptides Tc10 (insets **b1** and **b2**), At02 (insets **c1** and **c2**) and Cs02 (insets **d1** and **d2**) in the main phase transition of DMPC and 2:1 DMPC:DMPG LUVs, respectively, are also depicted.

**Figure 2:**
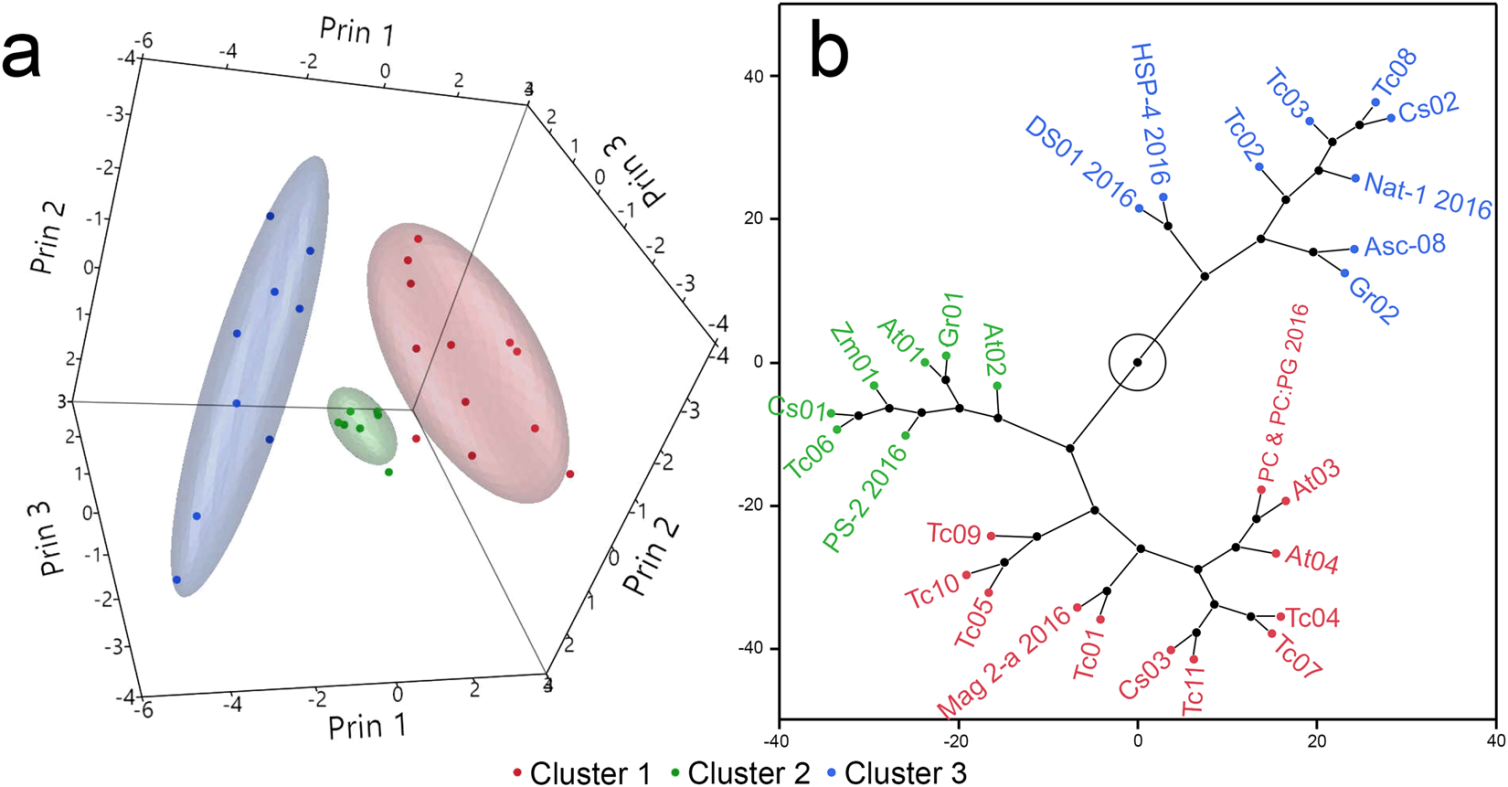
Multivariate statistics reveals similarities in peptide-induced perturbation of the main phase transition of model membranes probed by differential scanning calorimetry. A matrix describing the effect of peptides (Table 1, 2016 set) in the transition temperature (Tm), enthalpy (ΔH) and cooperativity (ΔH_VH_) of the broad and sharp components of DMPC and 2∶1 DMPC:DMPG LUV thermograms was assembled and standardized (Supplementary material 01). A principal component analysis was applied to the resulting data and the first five principal components, which explain ~87% of the data variance, were recorded (Supplementary material 01). A hierarchical clustering analysis (HCA) algorithm was applied to peptide locations in the first five principal components. (**a**) 3-D scatterplot of the peptide positions on the first three principal components. Peptide clusters, obtained from HCA, are color coded: Cluster 1, in red, cluster 2, green, cluster 3, blue. Normal contour ellipsoids (coverage 0.66) were calculated for peptide clusters. (**b**) Constellation plot of the dendrogram obtained from HCA categorizes peptides in discrete clusters. Cluster identities are represented by the same color code.

DSC scans of DMPC and 2:1 DMPC:DMPG LUVs enriched with 4 mol% peptides were performed and analyzed as previously reported^4,7^. Twenty-one novel putative IAPs were evaluated along with six AMPs, used as reference (Table 1, 2016 dataset). As is typical for such lipid vesicle systems, the DSC thermograms showed endothermic transitions characteristic of the phospholipid (P′_β_→L_α_) phase transition, usually comprising two broadly overlapping peaks^4,10^. The IAPs produced variable degrees of membrane perturbation, as illustrated in Fig. 1. The transition temperature (Tm), enthalpy (ΔH) and the van’t Hoff enthalpy (ΔH_v_) of the broad and sharp components of the main phase transitions of LUVs in the presence of peptides were extracted. To further explore these data, a principal component analysis was applied (PCA1), resulting in 5 principal components, which jointly explain ~87% of the data variance (Supplementary material 01). A scatterplot of the first three principal components was used to investigate similarities in the peptide-induced thermotropic behavior of the LUVs (Fig. 2a). A hierarchical clustering analysis algorithm was applied to the first five principal components of PCA1, resulting in three major peptide clusters, as observed in the constellation plot in Fig. 2b. Peptide clusters 1, 2 and 3 are depicted in red, green and blue, respectively, and normal contour ellipsoids were created in the PCA analysis to match peptide clusters, using the same color code (Fig. 2a). Cluster 1 encompasses peptides that induced minor changes in the main phase transition of DMPC LUVs, as judged by DSC data. However, in the case of 2:1 DMPC:DMPG LUVs, peptides from this cluster did give rise to rather more perturbation. This is exemplified by the peptides At03 and Tc10, shown in Fig. 1, insets a1, a2 and insets b1 and b2, respectively. This cluster comprises ten of the novel putative IAPs considered in this study and the AMP Mag-2a (Fig. 2b).

**Table 1:** Peptide names, ID, primary structures and their corresponding data set.

Peptide name	Peptide ID	Primary structure*	Dataset	References
Tc01	EOX93337.1 (95–110)	VALRLAKEVIKVQQGW	2016	^7^
Tc02	EOX91068.1 (202–221)	GKILKYLLYLLRKYANLIIR	2016	^7^
Tc03	Tc05_g014340 (12–32)	IKLRNVLKYLFRIDVIKEDIL	2016	^7^
Tc04	EOY21465.1 (193–214)	RVLKDVESALRESVANWKIVIG	2016	^7^
Tc05	EOY07102.1 (47–66)	IVNHLVKLFDKGLNSIVNLR	2016	^7^
Tc06	EOY24636.1 (117–135)	GSLHGFMYKYLKNMVLNLF	2016	^7^
Tc07	EOY07477.1 (79–98)	LIKVVNHVQYNVTLHWHGIR	2016	^7^
Tc08	EOY11557.1 (289–310)	LHRLVKLVAALLRGYASKVDTH	2016	^7^
Tc09	EOY11242.1 (688–707)	GIVLKDLFSEKLRRYKIVIG	2016	^7^
Tc10	EOY11257.1 (701–719)	GLLFKELQKLIRYQIFIGK	2016	^7^
Tc11	EOY17584.1 (39–55)	LLDKLKRTLLSIEAVLI	2016	^7^
At01	NP_172734.2 (118–136)	GSLHGFMYKYLKNMVLTLF	2016	^7^
At02	SIP121-0-PQ4 (45–60)	KVLSKVHTLLKAVLAL	2016	^7^
At03	NP_176750.1 (209–226)	GAKLAKKQVRALGKFFSF	2016	^7^
At04	NP_176750.1 (334–353)	GLYNFIKVLGRTVFGLYKQF	2016	^7^
Cs01	XP_006477592.1 (117–135)	GSLHGFMYRYLKNMVLNLF	2016	^7^
Zm01	XP_008663476.1 (153–171)	GSLHGFMYKYLKTLVLRLY	2016	^7^
Cs02	YP_740502.1 (463–480)	FFGHIWHGARTLFRDVFA	2016	^7^
Cs03	KDO49351.1 (129–146)	FFYNVIKIYGNMAGRISK	2016	^7^
Gr01	XP_012439503.1 (6–21)	GFKLGRKLVKVFKWII	2016	^7^
Gr02	KJB26672.1 (254–272)	ANRLLEAYKMLLKFLGNLR	2016	^7^
Ascaphin 8 (Asc-8)	—	GFKDLLKGAAKALVKTVLF	2016	^7,15^
Dermaseptin DS01 (DS01)	—	GLWSTIKQKGKEAAIAAAKAAGQAALGAL	2012 & 2016	^4,12^
Hylaseptin-4 (HSP-4)	—	GIGDILKNLAKAAGKAALHAVGESL	2012 & 2016	^4^
Magainin-2a (Mag-2a)	—	GIGKFLHSAKKFGKAFVGEIMNS	2012 & 2016	^4,13^
Nattererin-1 (Nat-1)	—	GLKDMIKNLAKEAAVKLAGAVINKFSPQPQ	2012 & 2016	^4,14^
PS-2	—	FLSLIPHAINAVSTLVHHF	2012 & 2016	^4,16^
P61458(35–60)	P61458(35–60)	FKQFHFKDFNRAFGFMTRVALQAEKL	2012	^4^
B0CZJ3(104–130)	B0CZJ3(104–130)	IAAAQRITSGAADIAINWAGGLHHAKK	2012	^4^
A4HW34(187–217)	A4HW34(187–217)	LVQRFHAYLHKFREAFMNVGAAAAVEGTKAA	2012	^4^
Q8RW88(70–95)	Q8RW88(70–95)	GHRGALKDWVQAAGGAVAAFDFTTKG	2012	^4^
Q6TV81(25–52)	Q6TV81(25–52)	AAAAAAAIKMLMDLVNERIMALNKKAKK	2012	^4^
O43312(33–62)	O43312(33–62)	FINKAGKLQSQLRTTVVAAAAFLDAFQKVA	2012	^4^
Q8KG25(327–351)	Q8KG25(327–351)	FVTNSKRLAEGIEKGVGNSILIKVN	2012	^4^
P94692(929–955)	P94692(929–955)	KLKKLLAGQKDGLLGQIAAMSDLYTKK	2012	^4^
B4FGE3(22–37)	B4FGE3(22–37)	KAGLQFPVGRIARFLK	2012	^4^
A3KLW0(117–136)	A3KLW0(117–136)	FKALRALRLEDLRIPTSYIK	2012	^4^
Q7YRI0(9–28)	Q7YRI0(9–28)	LAKRRVLTLLRQLRRVSPSS	2012	^4^
gb|ACU24018.1|(73–101)	gb|ACU24018.1|(73–101)	GLWQIFSSKEEGKDNSQQKSKGDQAKEL	2012	^4^
gb|AAD22970.1|(120–148)	gb|AAD22970.1|(120–148)	VWTTAMEKSSAANFSMSRNQRRSSLHSL	2012	^4^
Q9XEY7(120–148)	Q9XEY7(120–148)	SLWKNLSRKISGAVKAQPDLHTLLPLPGS	2012	^4^
A5LDU0(184–211)	A5LDU0(184–211)	GKFHQIKKMFLSVGVKVTSLKRIQFGDF	2012	^4^
DS 01 (1–12)	—	GLWSTIKQKGKE**	2012	^4^
Syphaxin	—	GVLDILKGAAKDLAGHVATKVINKI	2012	^4^
Pseudin B	—	GLNTLKKVIQGLHEVIKLVNNHA	2012	^4^
Hyposin HA-6	—	LRPAILVRVKGKGL	2012	^4^
Penetratin	—	RQIKIWFQNRRMKWKK	2012	^12,33^

*C-terminally amidated peptides.

**Free C-terminus.

Clusters 2 and 3 are composed of peptides that induced alterations in the main phase transition of LUVs of both compositions. The IAPs At02 and Cs02 are representative examples (Fig. 1, insets c1, c2 and insets d1, d2, respectively). The thermotropic behavior of model membranes in the presence of peptides from these groups are qualitatively similar, although cluster 3 molecules induced alterations characteristic of deeper insertions in the hydrocarbon core of model membranes^4,9^. Six IAPs constitute cluster 2 along with the AMP PS-2, while cluster 3 is composed of the AMPs DS 01, Nat-1, HSP-4, Asc-08 along with five novel IAPs (Fig. 2b).

### Perturbations in the main phase transition of LUVs correlate with antimicrobial activity

**Figure 3:**
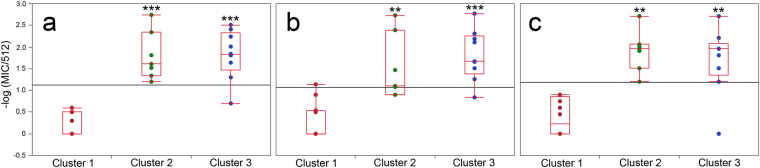
Peptide-induced perturbation of the main phase transition of model membranes is correlated to broad and potent antimicrobial activity. Minimum inhibitory concentration (MIC) assays were performed in biological triplicates for evaluated peptides (Supplementary dataset 01). MIC data were transformed to -log(MIC/512) and used to create a box plot as a function of peptide clusters obtained from the multivariate statistical analyses (Table 2). Microorganism classes were (**a**). Gram-positive bacteria, (**b**). Gram-negative bacteria, and (**c**). Yeasts. Please see text for a detailed description of the evaluated microorganisms. The Steel-Dwass all pairs statistical test was applied (**p < 0.01 or ***p < 0.001). Asterisks denote statistical difference of given clusters in relation to cluster 1. Horizontal grey lines correspond to the grand mean.

Antimicrobial activity was tested against three categories of human and plant pathogenic microorganisms: Gram-positive (*Bacillus subtilis, Bacillus cereus, Staphylococcus aureus*), Gram-negative (*Escherichia coli, Pseudomonas aeruginosa, Erwinia carotovora, Pseudomonas syringae* pv. *tabaci, Xanthomonas campestris* pv. *citri*) and yeasts (*Candida albicans, Cryptococcus neoformans*), as previously reported^7^. Clusters obtained above from similarities in peptide-induced alterations in main phase transition of model membranes are correlated to antimicrobial activity, as illustrated in Fig. 3 with data transformed to –log(MIC/512) for comparison. This data transformation ensures that peptides with negligible minimum inhibitory concentration (MIC) towards microorganisms (equal to or higher than 512 μM) will have zero or negative transformed activity. The full list of peptide MICs was published in an accompanying paper^7^, and is available in the current publication as supplementary dataset (Supplementary dataset 01). Median activities of peptide clusters for the three categories of microorganisms are listed in Table 2. Cluster 1 encompasses molecules with no detectable or just residual antimicrobial activity (Fig. 3). On the other hand, peptide clusters 2 and 3 presented significantly higher median antimicrobial activity than Cluster 1 peptides, regardless of the class of microorganisms under consideration (Kruskal-Wallis test, p < 0.001, Steel-Dwass All Pairs, α = 0.05, **p < 0.01 or ***p < 0.001). Nine IAPs distributed in clusters 2 and 3 presented MICs comparable to or lower than the reference AMPs considered in this study. These results demonstrate a high correlation between model membrane interaction and antimicrobial activity.

**Table 2:** Antimicrobial activity of peptides groups, transformed to -log(MIC/512), as a function of microorganism class.

	n	Gram-positive bacteria			Gram-negative bacteria			Yeasts		
		10%	Median	90%	10%	Median	90%	10%	Median	90%
Cluster 1	11	0	0	0.6	0	0	1.1	0	0.2	0.9
Cluster 2	7	1.2	1.6	2.7	0.9	1.10	2.7	1.2	2.0	2.7
Cluster 3	9	0.7	1.8	2.5	0.8	1.7	2.8	0	2.0	2.7

The 10%, 50% and 90% quantiles are shown.

### Differential scanning calorimetry as an incremental tool for the classification of membrane active peptides

**Figure 4:**
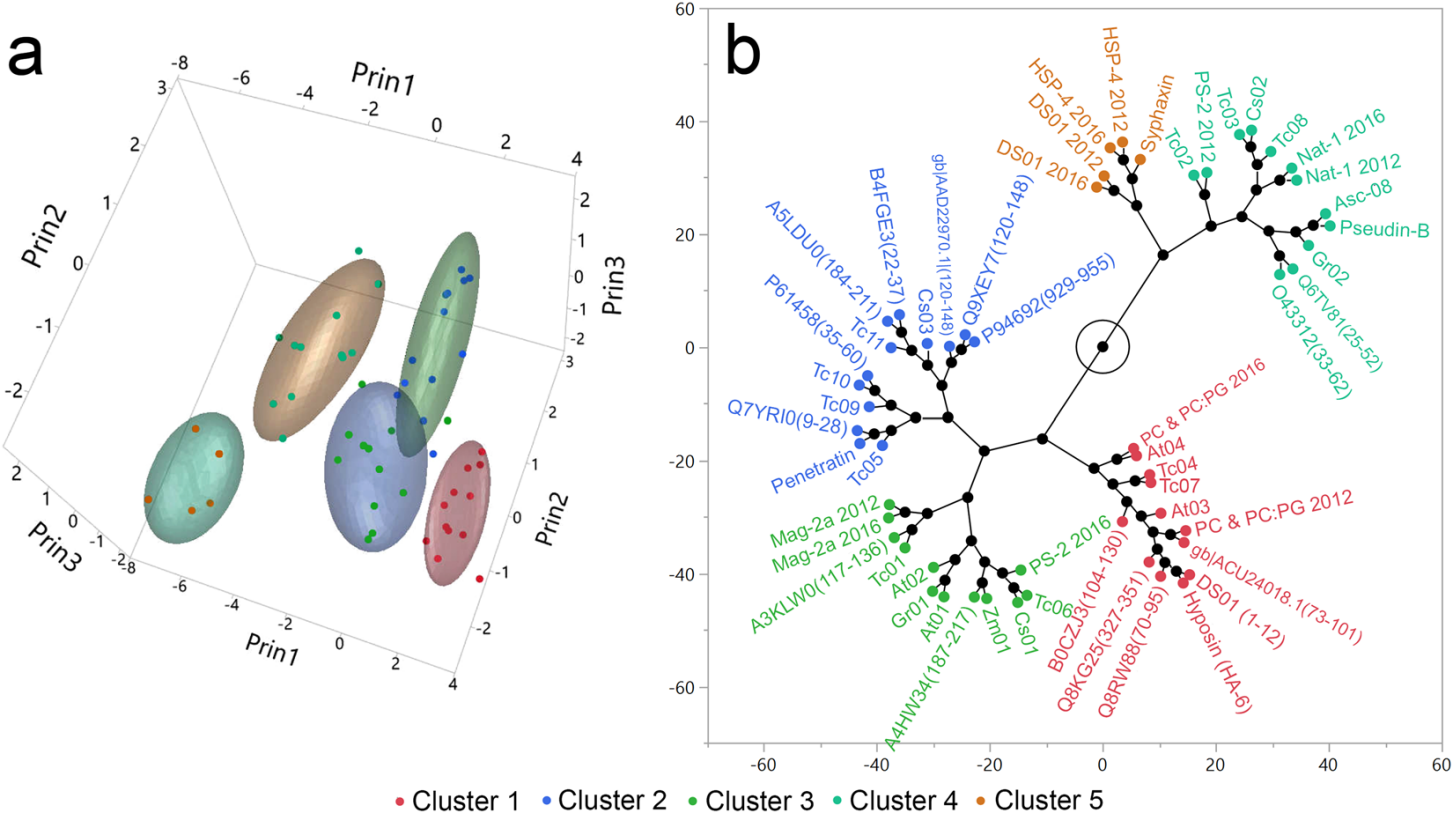
Meta-analysis of peptide-induced effects on thermograms can be used as an incremental framework for the classification of novel membrane-active peptides. Previously-acquired peptide-model membrane thermograms data^4^ were incorporated into the model (Table 1, 2012 and 2016 set). Peptides from the 2012 and 2016 data sets were standardized separately and submitted to a new principal component analysis (Supplementary material 02) and the first five principal components were recorded (~85% of the data variance). A hierarchical clustering analysis (HCA) algorithm was applied to peptide locations on the first five principal components. (**a**) 3-D scatterplot of the peptide positions on the first three principal components. Peptide clusters, obtained from HCA, are color coded: Cluster 1, in red, cluster 2 in blue, cluster 3 in green, cluster 4 in light green and cluster 5 in gold. Normal contour ellipsoids (coverage 0.66) were calculated for peptide clusters. (**b**) Constellation plot of the dendrogram obtained from HCA categorizes peptides in discrete clusters. Cluster identities are also represented using a color code.

Thermal scans obtained from the interaction of a previously evaluated set of IAPs and AMPs with model membranes were appended to current data for a meta-analysis^4^. Fifty-two peptides were considered, including duplicates of LUVs without any added peptide, and five duplicates of AMPs (DS01, Nat-01, HSP-4, PS-2 and Mag-2a) (Table 1, 2016 and 2012 datasets). Duplicate cases are identified with the year of data acquisition, either 2012 or 2016. Peptides from both datasets were standardized separately and jointly submitted to a separate principal component analysis, designated PCA2 (Supplementary material 02). The first three principal components were plotted (Fig. 4a) and a hierarchical clustering analysis algorithm was applied to the first five principal components of PCA2, resulting in the constellation plot depicted in Fig. 4b. Peptides were clustered in five groups. Clusters 1, 2, 3, 4 and 5 are represented as red, blue, green, light green and orange dots, respectively (Fig. 4a,b). Peptide duplicates from the 2012 and 2016 datasets were mostly confined to the same clusters, except for the AMP PS-2, as observed in Fig. 4b. Cluster 1 is composed by molecules with no significant interaction with the evaluated model membranes. Cluster 2 peptides present no detectable interaction with DMPC LUVs but disturb the main phase transition of 2:1 DMPC:DMPG LUVs. The peptide Penetratin is part of this cluster along with other IAPs. Clusters 3 and 4 are populated by IAPs and AMPs that alter the main phase transition DMPC and 2:1 DMPC:DMPG LUVs and present significant and broad antimicrobial activity, as demonstrated in the present study or in the literature^4^. The peptide cluster 5 is composed solely of AMPs (Fig. 4b).

### Membrane perturbation and peptide helix formation are correlated

**Figure 5:**
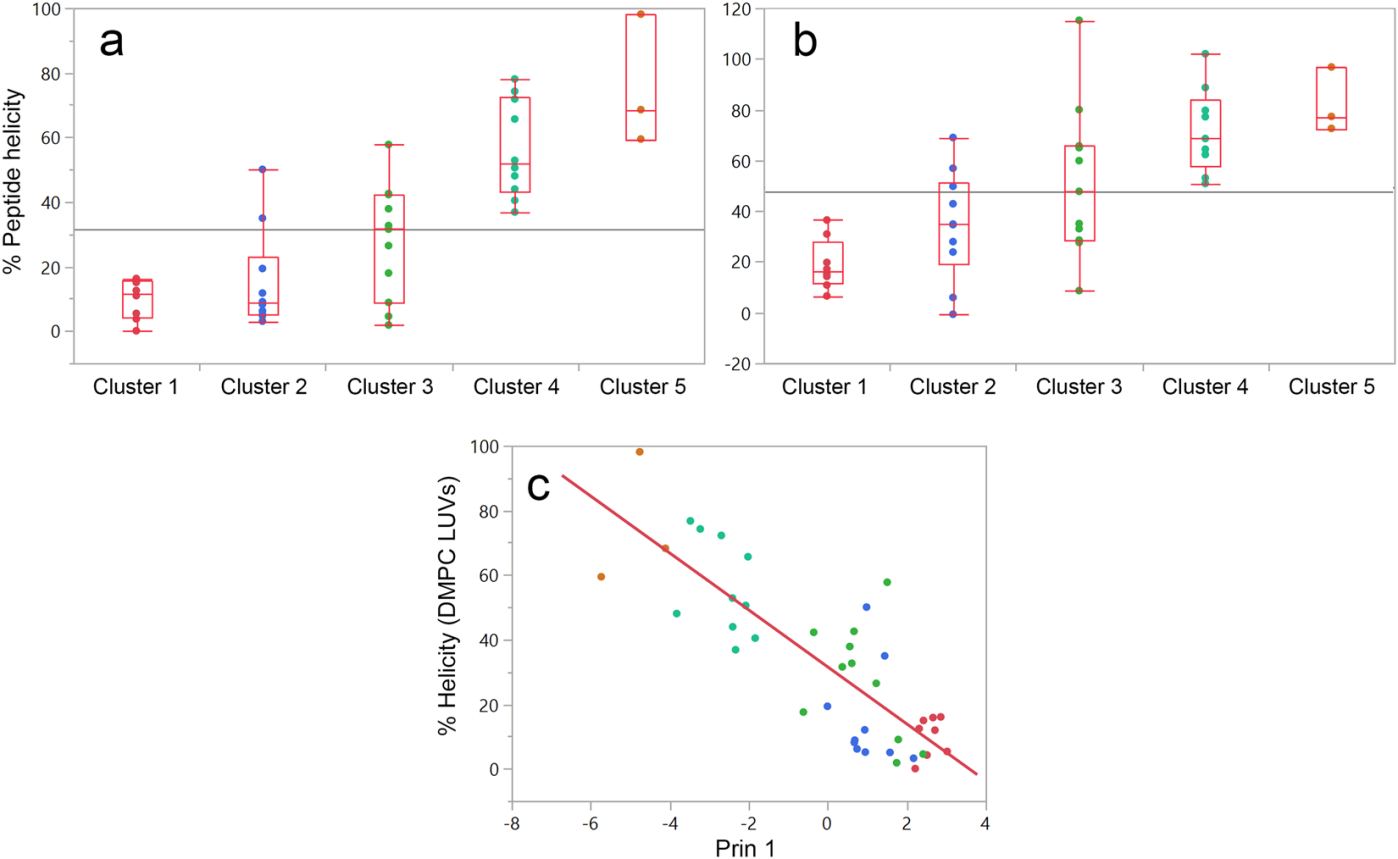
Peptide perturbations of model membranes are correlated to median percent helicity at 2 mol% peptide in DMPC and 2:1 DMPC:DMPG LUVs. Molar ellipticity of peptides at 222 nm was used to calculate peptide percent helicity in buffer and after the addition of LUVs at the specified molar ratio according to the method of Chen *et al*.^17^. A table was compiled (Supplementary material 03) and the median helicity of peptides after the addition of (**a**) DMPC and (**b**) 2:1 DMPC:DMPG LUVs was calculated as a function of cluster identity. (**c**) The relative position of peptides along the first principal component in PCA2 is linearly correlated to their percent helicity at 2 mol% in DMPC LUVs. The Pearson correlation between the relative position of peptides at Prin1 and their percent helicity when titrated with DMPC LUVs was r^2^ = 0.67, ANOVA p < 0.0001). Regression equation: Helicity (%) = 31 (±2)-9 (±1) × Prin1. Peptide clusters are color coded: Cluster 1, in red, cluster 2 in blue, cluster 3 in green, cluster 4 in green and cluster 5 in gold.

Far-UV CD scans were acquired for the twenty-one novel peptides in buffer alone and together with 2 mM DMPC or 2:1 DMPC:DMPG LUVs (2 mol% peptide/phospholipid) as reported in an accompanying paper^7^. These were appended to the corresponding data obtained for peptides on the extended dataset of molecules^4^ (Table 1, 2012 and 2016 datasets), and tabulated (Supplementary material 03). Duplicates were removed to prevent biases. Most peptides presented no regular structure in buffer alone. Nevertheless, a few molecules were already structured as β-sheets while others presented CD spectra compatible with partially structured α-helical segments. β-sheet peptides were excluded from further analyses. In general, the addition of LUVs to peptide solutions increased the α-helical content of molecules, regardless of model membrane composition. To quantify the conformational changes promoted by LUVs, data were transformed to the mean residue ellipcity (MRE) from which peptide percent helicity was estimated^4,17^. The correlation between peptide cluster identity, obtained from the PCA2 analysis, and their median percent helicity at 2 mol% peptide/phospholipid is depicted in Fig. 5a,b and listed in Table 3. Peptides from clusters 1 and 2 remained mostly unstructured after the addition of DMPC LUVs (Fig. 5a, Table 3). On the other hand, LUVs enriched with DMPG induced higher helical percentages for peptides from these clusters, especially for cluster 2 molecules. Peptide clusters 3, 4 and 5 show an increase in median helical percentages when mixed with LUVs (Table 3). As depicted in Fig. 5c, the first principal component is linearly correlated to the helical content of peptides in DMPC LUVs (n = 42 observations, R^2^ = 0.67, Analysis of Variance (ANOVA) p < 0.0001). This demonstrates that the extent of membrane perturbation is linearly correlated to peptide secondary structure, even for molecules without significant sequence similarity.

**Table 3:** Quantiles for peptide percent helicity in DMPC and 2:1 DMPC:DMPG LUVs as a function of peptide cluster.

Level	DMPC				2:1 DMPC:DMPG		
	n	10%	Median	90%	10%	Median	90%
Cluster 1	8	0	12	16	6	16	36
Cluster 2	10	3	8	48	1	34	68
Cluster 3	11	2	32	55	9	48	108
Cluster 4	10	37	52	76	51	66	99
Cluster 5	3	59	68	98	72	77	97

### Reassessment of the physicochemical properties of peptide clusters

**Figure 6:**
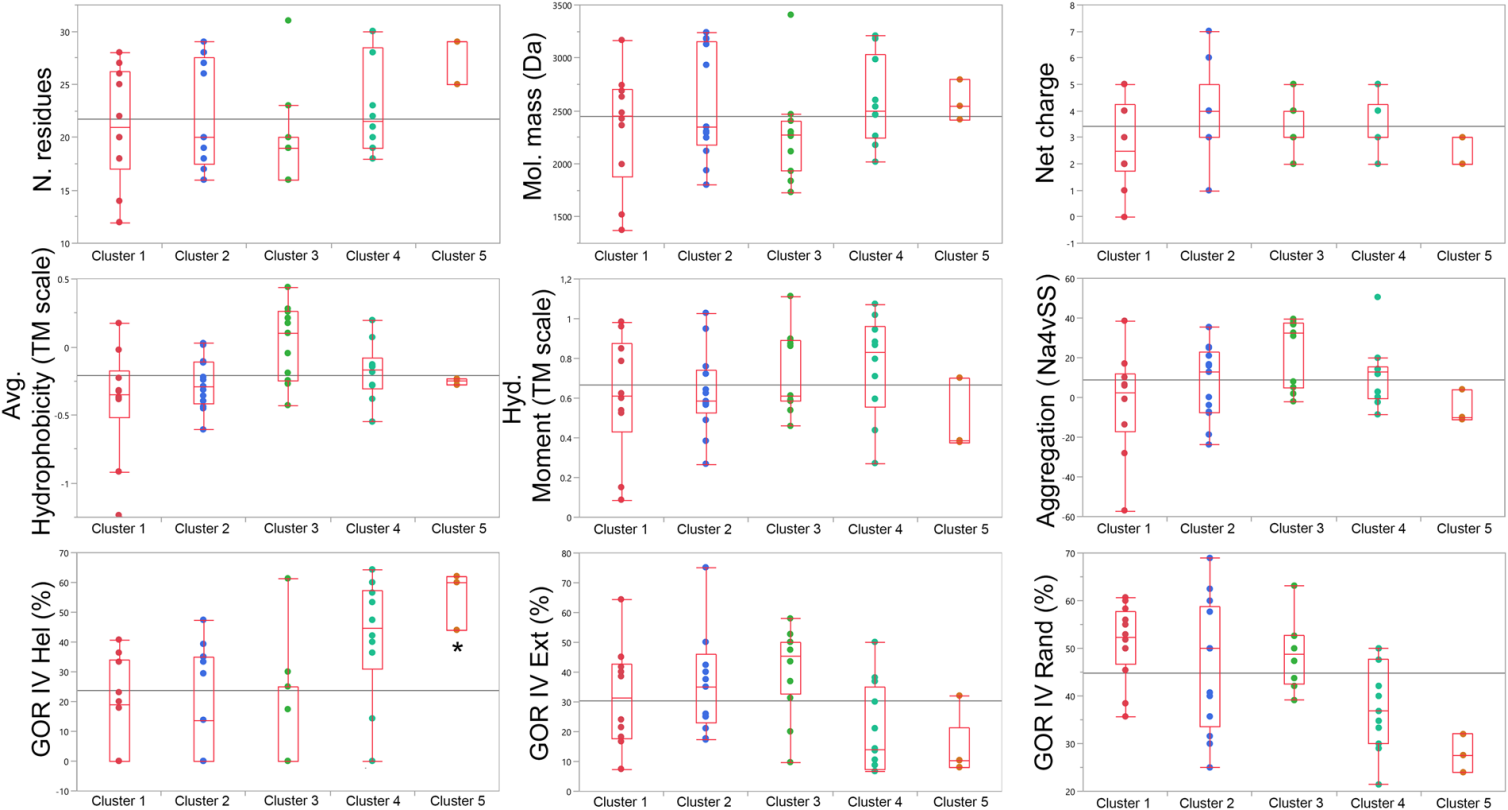
Box plot of peptide clusters and their corresponding physicochemical properties. The physicochemical properties of the extended set of peptides (Table 1, 2012 and 2016 sets) were calculated and plotted as a function of cluster memberships, as obtained from the PCA2 analysis. The residue number (N. residues), molecular mass (Mol. mass (Da)), net charge, average hydrophobicity (Avg. Hydrophobicity (TM scale)^1^, hydrophobic moment (Hyd. Moment (TM scale)), aggregation potential (Agreggation (Na4vSS))^18^, and secondary structure according to the GOR IV algorithm^20^, (GOR IV Helical, GOR IV Extended and GOR IV rand) were determined. The Steel-Dwass all pairs statistical test was applied (*p < 0.05). Please refer to Supplementary material 04 document for a complete description of statistical tests and their significances. Horizontal grey lines correspond to the grand mean.

Similarities in the peptide-induced changes to thermotropic profiles of model membranes implicate a similar set of peptide physicochemical properties with relevance to interfacial membrane activity. Therefore, the median physicochemical properties of the peptide clusters from PCA2 were reassessed (Supplementary material 04). Forty-eight peptides were analyzed, after removal of duplicate cases to prevent biases. The residue number, molecular mass, net charge, aggregation potential (Na4vSS parameter of the AggreScan algorithm)^18^, hydrophobicity (Transmembrane tendency (TM) scale)^1^, hydrophobic moment^19^ (using the TM scale) and secondary structure according to the GOR IV algorithm^20^ (which considers helical, extended and random structures), were evaluated as a function of peptide clusters (Fig. 6). As expected, there is a superposition in the properties of membrane active peptides clusters, leading mostly to non-significant median differences (Supplementary material 04). Indeed, no single distinctive physicochemical property emerges when peptides from Clusters 1 and 2, which present superficial membrane interactions (Fig. 2) and are non-antimicrobial (Fig. 3), are compared to the peptide clusters populated by actual antimicrobial molecules (Clusters 3, 4 and 5). Nevertheless, some tendencies in the physicochemical properties of peptides on clusters 3, 4 and 5 can be observed (Fig. 6). For these clusters, the number of amino acid residues is positively correlated with peptide cluster number. On the other hand, the average hydrophobicity and tendency to aggregate present the opposite pattern: cluster 5 peptides are less hydrophobic and are less likely to aggregate then cluster 3 peptides. Furthermore, the GOR IV algorithm predicts an increasing percent helicity in peptides from cluster 5 when compared to the remaining clusters. This data demonstrate that actual antimicrobial peptides present subtle but relevant differences in their physicochemical nature, and that these might be linked to their modes of membrane interaction.

## Discussion

Bioactive peptides encrypted in protein sequences appear to be much more frequent in nature than initially envisioned, and presently we are just beginning to grasp their importance as endogenous physiological regulators and/or their biotechnological potential^21,22^. It is not an overstatement to hypothesize that some proteins could be perceived as assemblies of various putative bioactive peptides joined head-to-tail, whose decryption might trigger a physiological response in a native or man-made scenario^4,7,23^. In other words, after the complete ribosomal synthesis, with proper polypeptide folding and post-translation modifications in place, the resulting mature protein could also be seen as a potential carrier of bioactive peptides that may be released under proteolysis or any other degradation process to meet desirable targets and, therefore, fulfill biological roles different from the original mature intact protein. Based on this rationale, we recently introduced a methodology to uncover encrypted intragenic antimicrobial peptides, prospected *in silico* from genomes regardless of the original structure/function of the parent proteins^4,7^. This procedure relies on the calculation of physicochemical properties of theoretical protein fragments in comparison to a sample of naturally occurring AMPs^4^. However, despite significant efforts, the discrimination of antimicrobial from non-antimicrobial peptides based solely in physicochemical descriptors is challenging^24^. It has been reported that AMPs, CPPs and PATs are structurally and functionally superposed classes, which result from the variation that each peptide displays in its tendency to bind, desorb, oligomerize, permeabilize, and translocate across a given membrane^2,25^. Considering that our methodology uses physicochemical properties as descriptors of peptide biological activity, putative IAPs uncovered by the software Kamal must present different qualities of membrane interaction. This work describes our latest efforts on the identification, characterization and refining of putative classes of membrane active peptides, with an emphasis on antimicrobial molecules.

Thermal transitions in model membranes enriched with peptide additives were examined by DSC and the P′_β_→L_α_ transitions were fit to a non-two state model (Fig. 1). Resulting data were analyzed using multivariate statistics, which revealed three distinct peptide clusters (Fig. 2). Significant and broad antimicrobial activities were associated with two clusters, both composed of peptides that disturbed the main phase transition of DMPC LUVs (Fig. 3). Peptides from these two clusters were active against all microorganisms tested, regardless of their particular membrane compositions (Fig. 3). This result indicates that the interaction of these peptides with DMPC LUVs is representative of those taking place in more complex membrane systems, and that the antimicrobial activity is more a property of the peptide than that of the target membranes, in consonance with the interfacial activity model^26^. IAPs from clusters 2 and 3 in Fig. 2a are thus active against a broad range of microorganisms, as are classical AMPs, and may also share other biological activities^27,28^.

Cluster 1 peptides disturbed the main phase transition of model membranes to a lesser extent and did so only when the negatively charged phospholipid DMPG was present. Nevertheless, these peptides were capable of inducing membrane microdefects and influence the Brownian motion of phospholipids, and therefore, possess interfacial activity^26^. The antimicrobial activities observed for cluster 1 peptides are relatively minor (Fig. 3), and can be regarded as consequence of less disruptive modes of peptide-membrane interaction. It is clear, however, that membranolytic peptides produce a prototypic thermal profile in model vesicles and that this can be used to categorize novel membrane active molecules with unknown biological activity.

These conclusions were reinforced by merging the current dataset to thermal transition data of model membranes enriched with a previous generation of IAPs and control AMPs (Table 1, 2012 dataset) jointly submitted to multivariate statistical analyses (Fig. 4). The incorporation of the 2012 dataset not only increases the diversity of evaluated peptides, but also tests the robustness of the model. Indeed, duplicates of model membranes:AMP interactions obtained in different laboratories at different times were in good agreement, as observed in Fig. 4b. Mag-2a, HSP-4, DS01 and Nat-1 scans obtained in 2012 and 2016 are neighboring branches in the constellation plot in Fig. 4b, while DMPC & 2:1 DMPC:DMPG are within the same cluster. This indicates good data reproducibility. The AMP PS-2, categorized in different clusters, is the only exception, and should be further investigated. Furthermore, the 3-D scatterplot of the PCA1 (Fig. 2a) and PCA2 (Fig. 4a) analyses share the same overall graph topology, a consequence of the high correlation between their principal components loadings (Supplementary material 01 and 02). It is worth mentioning that the space of putative peptide-membrane interactions is continuous and peptide cluster numbers and memberships are subject to variations as the model develops. Nevertheless, we propose that novel putative IAP-membrane interactions can be gradually incorporated, resulting in the classification of peptides with unknown modes of membrane interaction, and the enrichment of the predictive framework.

The data presented herein also point to a correlation between the thermotropic effect of peptides on membranes and peptide helix formation, as depicted in Fig. 5. Peptide clusters 1 and 2 of the PCA2 analysis remained mostly unstructured upon the addition of LUVs, while peptide clusters 3, 4 and 5 transitioned to more helical structures (Fig. 5a,b). Indeed, Prin1, the first principal component of the PCA2, is correlated with peptide percent helicity in DMPC LUVs (Fig. 5c). These results concur with the observation that binding of peptides at lipid bilayers of reduced negative surface charge is strongly modulated by the tendency toward helix formation^29^. A quantitative (linear) link between far-UV CD measurements and thermal scans of peptide:membrane mixtures is demonstrated. A first attempt to quantify the effect of peptides on the phase transition of DMPC LUVs and their helicity in the same environment is given by the equation (standard errors are in brackets):

1 $${\rm{Helicity}}\,( \% )=31\,(\pm 2)-9\,(\pm 1)\times {\rm{Prin}}1$$

Where Prin1 is the peptide location in the first Principal Component of the Principal component analysis plotted in Fig. 4. Equation 1 can be used to probe the depth of peptide insertion in the acyl core of membranes, its capacity to disturb membrane-stabilizing hydrophobic lipid interactions and consequently evaluate its antimicrobial potency towards microorganisms.

It is implicit in our methodology that similar modes of peptide-membrane interaction reflect similar set of peptide physicochemical properties. To investigate this assertion, physicochemical properties of peptide clusters were calculated from their primary structure (Supplementary material 04). A considerable overlap in the physicochemical properties of peptide clusters was detected, as depicted in Fig. 6. There are, however, distinctive properties for molecules on clusters 3, 4 and 5, indicating that antimicrobial peptides are indeed a heterogeneous group. It is well established that antimicrobial peptides permeate membranes by different mechanisms^11,26^. However, a direct link between peptide physicochemical properties and modes of membrane interaction has not been explicitly postulated until now. Considering the theoretical features and the experimental data presented herein, it is possible to hypothesize that cluster 3 peptides are driven towards membranes due to their high average hydrophobicity (Fig. 6). However, these molecules are relatively small (Fig. 6, Nr. residues) and only partly structured as α-helical segments in LUVs (Table 3, Fig. 3) and therefore reside in superficial locations and do not disturb the main phase transition of membranes so significantly. The peptide PS-2, isolated from the skin secretion of the amphibian *Phyllomedusa azurea*^16^ is an AMP that represents this group (Fig. 4b, PS-2 2016). On the other hand, peptide clusters 4 and 5 are composed of molecules with longer polypeptide chains (Fig. 6, Nr. residues) that have the tendency to structure more significantly into α-helical segments (Fig. 3) and therefore disturb the inner hydrocarbon interior of membranes. Peptides from clusters 4 and 5 diverge only in the extent to which they perturb the acyl interior of model membranes, and do not present differential physicochemical properties (Fig. 6). It is however intuitive that cluster 5 molecules might present a lower hydrophobic moment than cluster 4 peptides, and that the former should be physicochemically more similar to transmembrane protein segments than the latter^1,30^. Nattererin-1, from *Physalaemus nattereri*^4,14^, represents cluster 4 peptides, and DS01^12^, first isolated from the amphibian *Phyllomedusa oreades*, represents cluster 5. The high helical propensity of peptide clusters 4 and 5 in neutral membranes is apparently captured by the GORIV algorithm (Fig. 6). The strength of this association must be further verified by the incorporation of additional novel molecules to the model. Nevertheless, it is fortuitous that an algorithm originally designed for the prediction of secondary structures can be helpful in the uncovering of potent antimicrobial peptides.

Finally, the experimental data reported here derived from peptide/phospholipid interactions obtained in ideal physical-chemical conditions, a fully *in vitro* quantitative and reproducible methodology, were remarkably related to microbicidal activities against pathogenic bacteria and fungi using the same peptides investigated in present study. A matching set of data exploring the biological relevancies and presenting the MIC values for each one of these molecules is reported in the accompanying paper^7^.

## Methods

### Peptides

Details on the peptides used in the present study, such as the Kamal algorithm filtering parameters, the methodology for solid phase peptide synthesis, high performance liquid chromatography (HPLC) purification, confirmation by mass spectrometry, and peptide quantification, are reported in “Encrypted Antimicrobial Peptides from Plant Proteins”^7^. Peptides are identified by their publication names, by their UniProtKB or Genbank (gb|) accession number followed by the indication of the first and last amino acid residues in brackets.

### Lipid vesicles

DMPC and DMPG were purchased from Avanti (Avanti Polar Lipids, AL, USA). DMPC and 2:1 DMPC:DMPG (w/w) were dissolved in chloroform and methanol (3:1 v/v) at 10 mg/mL, dried using a rotary evaporator and left 3 hours under high vacuum in a freeze-drier. Phospholipids were then dispersed in 20 mM sodium phosphate-NaOH, 150 mM NaCl, pH 7.4, and hand-shaken until the formation of a cloudy suspension, which was passed 19 times through a 100 nm polycarbonate membrane at 30 °C for the formation of LUVs using a mini-extruder (Avanti Polar Lipids, AL, USA). Phospholipid concentration was estimated according to the ammonium ferrothiocyanate method^31^.

### Differential scanning calorimetry (DSC)

Thermograms were obtained using a VP-DSC (MicroCal Inc., MA, USA) over a temperature range from 10 to 40 °C at a scanning rate of 0.5 °C/min. Blank thermograms using buffer alone and 0.5 mM DMPC or 2:1 (w/w) DMPC:DMPG LUVs in buffer were acquired as reference. Peptides were added to fresh samples of 0.5 mM LUVs at a concentration of 20 μM (0.04 mol/mol peptide/phospholipids) at room temperature, immediately followed by DSC data acquisition. Each sample was subjected to repeated thermal scans until there were no distinguishable changes in the thermal profile of the main phase transition (P′_β_→L_α_) of phospholipids between scans. Data were concentration normalized, baseline subtracted (linear connect), and fit to a non two-state transition for two peaks using MicroCal Origin software v7.0. Re-scans for selected cases were acquired using fresh peptide and LUVs solutions to check the reproducibility of the data.

### Circular Dichroism

Experiments were conducted on a Jasco-J815 spectropolarimeter (Jasco International Co., Japan). Spectra were acquired at room temperature from 200 to 260 nm as an average of 4 readings using a 0.1 cm path length cell, data pitch of 0.2 nm and a response time of 0.5 s. Scans of buffer and 2 mM DMPC and 2:1 DMPC:DMPG LUVs solutions were subtracted from each peptide data. Peptides were scanned at a concentration of 40 μM in buffer and then 50 fold excess of DMPC or 2:1 DMPC:DMPG LUVs was added, resulting in a molar ratio of 0.02 peptide/phospholipid. The spectra were converted to mean residue ellipticity and readings at [θ]_222_ nm were used to estimate helix percentages^17^.

### Inhibitory activity of IAPs

The determination of the minimum inhibitory concentration (MIC) of peptides was performed as described in “Encrypted Antimicrobial Peptides from Plant Proteins”^7^. Briefly, MICs were determined following protocols M7-A10 and M27-A3 from Clinical & Laboratory Standards Institute (CLSI) for bacteria and yeasts, respectively. Different concentrations of IAPs (0.5 to 256 μM) were tested against several microorganisms in a final volume of 100 µL. Three biological repetitions, with 2 technical replicates each, were performed for each test, on polystyrene flat-bottom 96 wells microplates. The minimum inhibitory concentration (MIC) was defined as the concentration at which no cells were detected when visualized by optical microscopy. Please refer to the literature^7^ for further details of the microbiological assays.

### *In silico* calculation of peptide physicochemical properties

The net charge, molecular mass, average hydrophobicity, hydrophobic moment^19^, aggregation potential (Na4vSS property of the the AggreScan algorithm^18^) and peptide secondary structure (according to the GOR IV algorithm^20^) were calculated for each molecule using an add-on for the software Kamal v1.0 *alpha*^4^. The TM scale was employed in hydrophobicity calculations^1^. Isoelectric point was calculated according to pK values extracted from the literature^32^.

### Statistical analyses

Exploratory data analysis and statistical tests were performed using the JMP^®^ Version 13 software (SAS Institute Inc., NC). Experimental data were standardized previous to Principal Component Analyses. For the PCA2 analysis, data standardization was performed independently on the 2012 and the 2016 data sets to compensate for analytical variations in data acquisition. The first five principal components of the PCA1 and PCA2 analyses were submitted to a hierarchical clustering algorithm using the Ward´s method without a second data standardization. The number of clusters in hierarchical clustering analysis was chosen arbitrarily. Non-parametric comparisons for all pairs using the Steel-Dwass method were also calculated using the JMP software.

### Data availability

All data generated or analyzed during this study are included in this published article (and its Supplementary Information files).

## Electronic supplementary material

Supplementary material

Supplementary Dataset 01
