# Supplementary material for: Towards an experimental classification system for membrane active peptides
Source: Sci Rep. 2018 Jan 19;8:1194. doi: 10.1038/s41598-018-19566-w (PMC5775428; doi:10.1038/s41598-018-19566-w)

Towards an experimental classification system for membrane active peptides

Brand, G.D.[1,2], Ramada, M.H.S.[1,3,4], Genaro-Mattos, T.C.[5], Bloch, C. Jr.[1,*]

[1] Laboratório de Espectrometria de Massa, Embrapa Recursos Genéticos e Biotecnologia, Brasília – DF, Brazil
[2] Laboratório de Síntese e Análise de Biomoléculas, Instituto de Química, Universidade de Brasília, Brasília – DF, Brazil
[3] Departamento de Biologia Celular, Instituto de Ciências Biológicas, Universidade de Brasília, 70910-900, Brasília, DF, Brazil.
[4] Pós-Graduação em Ciências Genômicas e Biotecnologia, Universidade Católica de Brasília, 70790-160, Brasília, DF, Brazil.
[5] Chemistry Department, Vanderbilt University, Nashville – TN, USA.

Supplementary material

Supplementary Material 01

Table S1.1. Numerical data resulting from the fitting of the P´β→Lα phase transition of DMPC and 2:1DMPC:DMPG LUVs added with peptides to a non-two state model with two manually assigned peaks.

	Tm_broad_pc	ΔH_broad_pc	ΔHv_broad_pc	Tm_sharp_pc	ΔH_sharp_pc	ΔHv_sharp_pc	Heat_pc	Tm_broad_pcpg	ΔH_broad_pcpg	ΔHv_broad_pcpg	Tm_sharp_pcpg	ΔH_sharp_pcpg	ΔHv_sharp_pcpg	Heat_pcpg
PC & 2:1														
PC:PG	23.54	2743	236400	24.13	2634	664900	5452	23.85	2246	202700	24.25	3290	624300	5656
PS-2	24.37	3608	100900	23.03	1988	442600	5536	24.65	2898	125000	24.01	2606	587500	5522
DS01	24.99	1654	154000	21.59	318.7	204000	1860	25.93	1979	89700	20.05	321.3	172400	2233
Tc02	23.55	5200	84750	21.94	1461	272000	6495	24.09	2107	72360	19.05	2018	210300	4039
Tc01	23.32	3988	202000	23.94	2392	649000	6485	24.50	3820	112700	22.81	2091	397900	5835
Tc03	24.18	4023	113500	23.06	1674	349000	5571	24.86	2096	115200	20.02	820.2	129200	2862
Tc04	23.29	3780	190000	23.93	2686	607000	6621	24.62	1946	169900	23.90	2384	690400	4297
Tc05	24.30	4550	154000	23.43	2585	658000	7243	26.30	2100	122400	23.41	3387	390200	5546
Tc06	24.76	3173	103500	21.91	2862	599700	5978	24.91	2727	113800	23.43	2599	563300	5288
Tc07	23.28	4043	199800	23.94	2363	664100	6604	24.35	2360	150100	24.28	2345	602900	4701
Tc08	24.25	4441	102000	23.03	2192	425000	6578	25.65	1774	112800	21.24	1164	124000	2930
Tc09	23.11	4203	175800	23.74	2429	599000	6859	26.54	1560	110900	24.29	1083	261400	2650
Tc10	23.20	4147	177000	23.68	2638	539000	6998	25.07	2444	110100	20.96	2520	165200	4963
Tc11	23.59	3818	109500	23.68	2201	514800	6088	24.89	2151	118200	24.51	2118	632200	4306
At01	24.92	3023	110400	22.96	3139	695400	6079	25.32	2794	106700	24.17	2978	571400	5748
At02	25.10	2915	125000	23.35	3114	500300	6052	24.29	3475	77010	21.87	2296	576300	5670
At03	23.71	2536	161200	23.97	2975	495000	5545	24.57	2508	159100	24.08	2672	683400	5195
At04	23.78	3819	257000	24.11	1297	799000	5476	23.93	2896	154200	24.14	2201	602000	5217
Asc-8	24.79	2456	120800	20.84	3254	152300	5626	24.98	1825	110900	19.62	1332	219000	3136
Cs01	24.98	2988	109900	22.35	2806	658800	5764	25.27	2667	112900	23.72	2835	575500	5452
Zm01	24.92	3067	102700	22.58	2477	529900	5530	25.36	2502	101400	24.59	2181	491200	4624
Cs02	23.78	4605	75410	22.15	1040	386100	5433	26.00	2655	107000	21.49	1205	193300	3905
Cs03	23.68	3160	223000	24.08	2199	643900	5550	24.83	2195	114500	23.92	2233	483100	4405
Gr01	25.19	2530	137600	23.13	3206	604000	5763	25.18	3178	111500	25.21	2839	612300	6082
Gr02	25.09	3574	91960	22.06	772	310200	4271	24.93	2987	105100	21.34	2606	313200	5518
Mag-2a	23.58	2759	191700	23.93	2773	555900	5649	24.06	4600	96010	22.87	1100	449100	5620
HSP-4	25.87	2339	112300	22.28	1010	160800	3318	26.31	2230	104500	21.80	899	116600	3112
Nat-1	24.86	4197	90360	22.57	893.6	320300	5024	26.05	2164	105900	21.26	271.2	144500	2421

Legend:
Tm = transition temperature, given in °C
Broad = broad component of the transition
Sharp = sharp component of the transition
Heat = total area under de curve
Pc = DMPC LUVs
Pcpg = 2:1 DMPC:DMPG LUVs
ΔH = transition enthalpy, given in $kcal.mol^{-1}$
ΔHv = van´t Hoff transition enthalpy, given in $kcal.mol^{-1}$

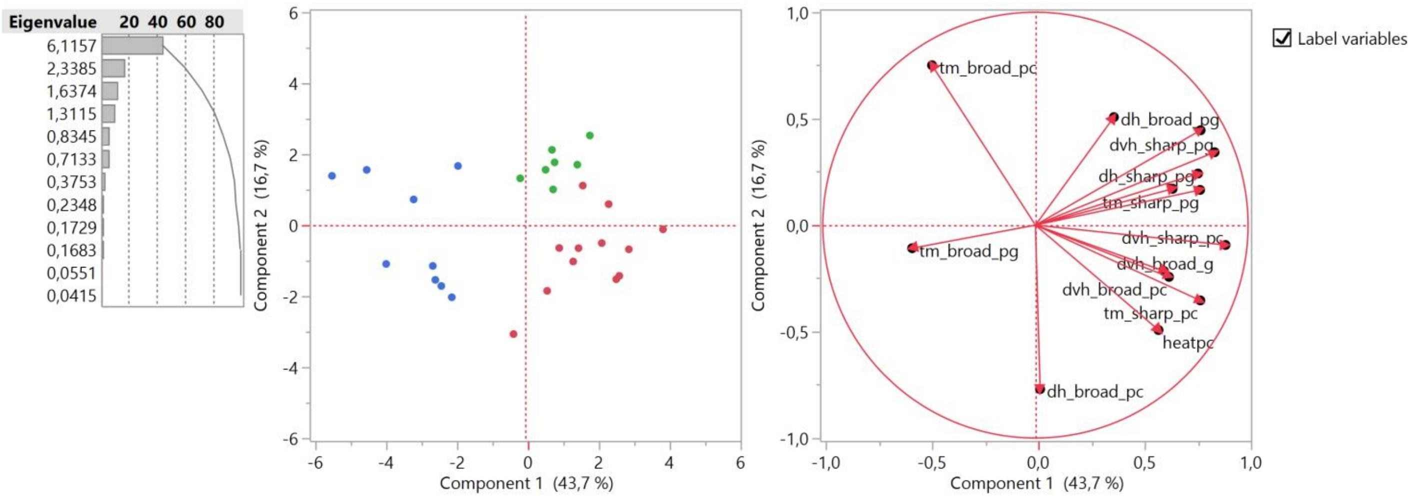

Figure S1.1. Summary plot of the PCA1. Cluster 1, 2 and 3 peptides are represented as red, green and blue dots, respectively.

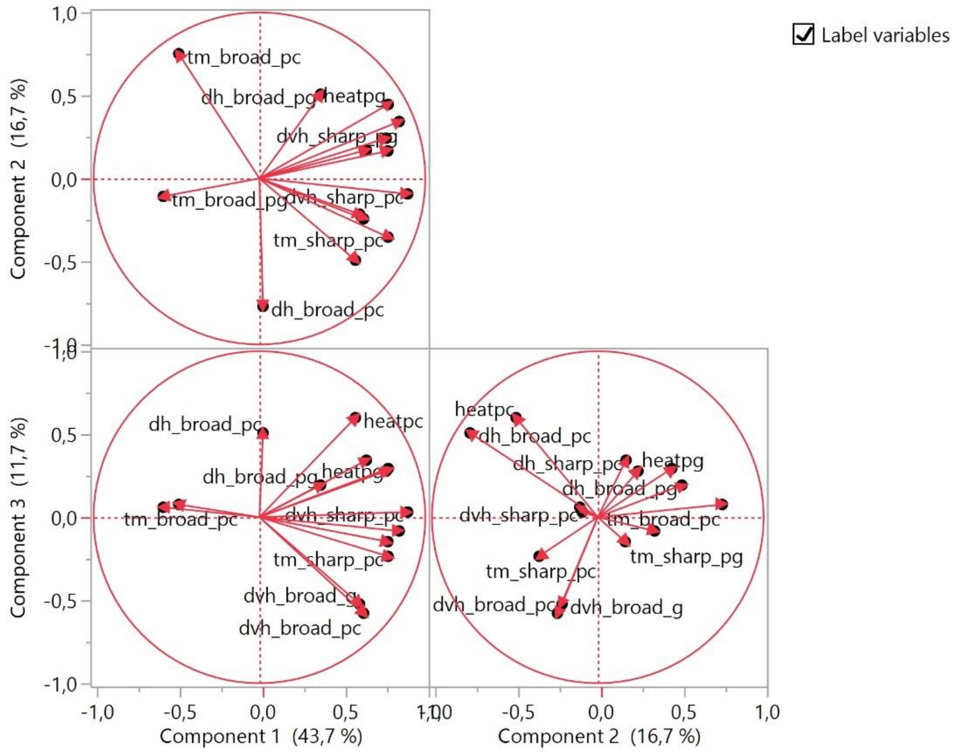

	Prin1	Prin2	Prin3	Prin4	Prin5
tm_broad_pc	-0,48630	0,75072	0,07622	0,26796	0,06384
dh_broad_pc	0,02191	-0,77013	0,50730	-0,09416	0,05829
dvh_broad_pc	0,62797	-0,24322	-0,57830	-0,21451	0,07526
tm_sharp_pc	0,77534	-0,35371	-0,23555	-0,12240	0,29058
dh_sharp_pc	0,64496	0,17044	0,34309	0,24414	-0,16564
dvh_sharp_pc	0,89384	-0,09337	0,03109	0,11347	0,28503
heatpc	0,57860	-0,49265	0,59924	0,10605	-0,04734
tm_broad_pg	-0,57978	-0,10845	0,05972	0,59340	0,48034
dh_broad_pg	0,36892	0,50716	0,19315	-0,64029	0,33318
dvh_broad_g	0,60383	-0,21539	-0,52349	0,24705	-0,32326
tm_sharp_pg	0,77416	0,16525	-0,14747	0,40091	0,32997
dh_sharp_pg	0,76436	0,24216	0,27747	0,22669	-0,24808
dvh_sharp_pg	0,84254	0,34245	-0,08332	0,14580	-0,09641
heatpg	0,77665	0,44554	0,29317	-0,17212	0,00365

Figure S1.2. Loading plot of the first three principal components of the PCA1 analysis and loading matrix of the first five principal components.

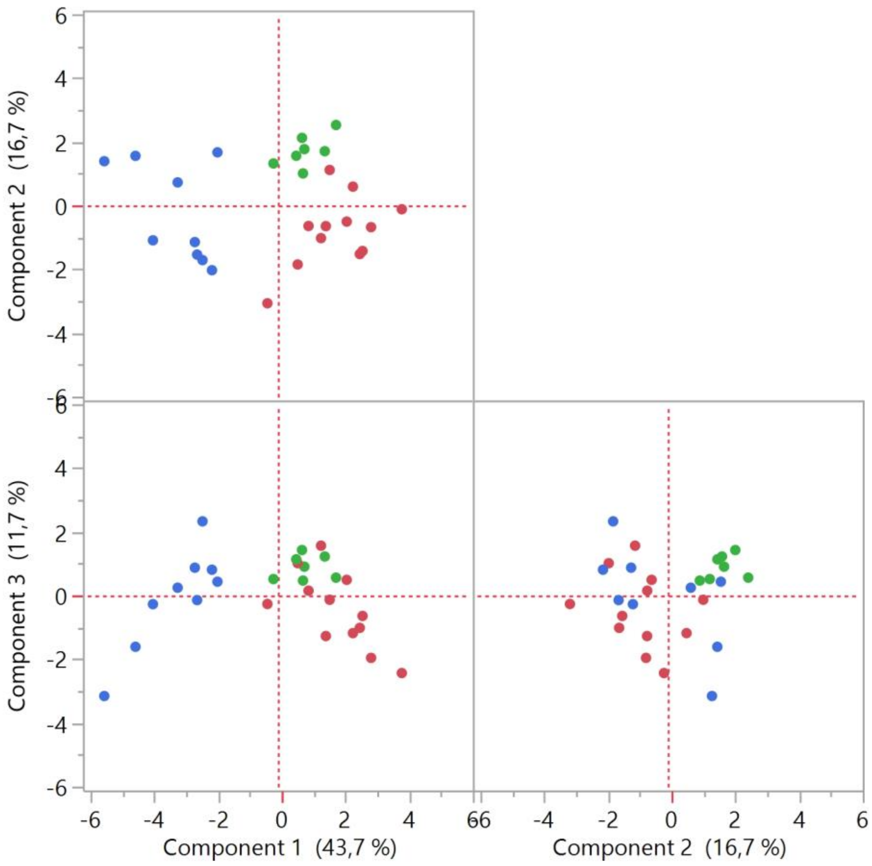

Figure S1.3. Score plot of the first three principal components of the PCA1. Cluster 1, 2 and 3 peptides are represented as red, green and blue dots, respectively.

Number	Eigenvalue	Percent		Cum Percent
1	6,1157	43,683		43,683
2	2,3385	16,704		60,387
3	1,6374	11,696		72,083
4	1,3115	9,368		81,451
5	0,8345	5,960		87,411
6	0,7133	5,095		92,506
7	0,3753	2,680		95,186
8	0,2348	1,677		96,863
9	0,1729	1,235		98,098
10	0,1683	1,202		99,301
11	0,0551	0,393		99,694
12	0,0415	0,296		99,990
13	0,0008	0,006		99,996
14	0,0005	0,004		100,000

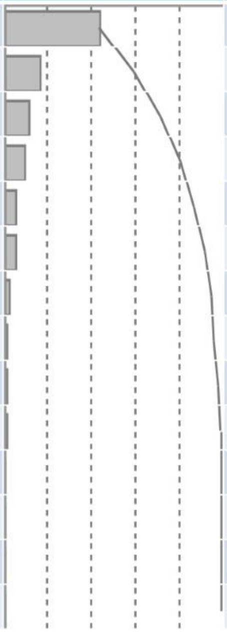

Figure S1.4. Eigenvalues of the PCA1 analysis.

Supplementary Material 02

Table S2.1. Numerical data resulting from the fitting of the P´β→Lα phase transition of DMPC and 2:1DMPC:DMPG LUVs added with peptides to a non-two state model with two manually assigned peaks.

	Tm_broad_pc	ΔH_broad_pc	ΔHv_broad_pc	Tm_sharp_pc	ΔH_sharp_pc	ΔHv_sharp_pc	Heat_pc	Tm_broad_pcpg	ΔH_broad_pcpg	ΔHv_broad_pcpg	Tm_sharp_pcpg	ΔH_sharp_pcpg	ΔHv_sharp_pcpg	Heat_pcpg
PC & 2:1 PC:PG	23.54	2743	236400	24.13	2634	664900	5452	23.85	2246	202700	24.25	3290	624300	5656
PS-2	24.37	3608	100900	23.03	1988	442600	5536	24.65	2898	125000	24.01	2606	587500	5522
DS01	24.99	1654	154000	21.59	318.7	204000	1860	25.93	1979	89700	20.05	321.3	172400	2233
Tc02	23.55	5200	84750	21.94	1461	272000	6495	24.09	2107	72360	19.05	2018	210300	4039
Tc01	23.32	3988	202000	23.94	2392	649000	6485	24.50	3820	112700	22.81	2091	397900	5835
Tc03	24.18	4023	113500	23.06	1674	349000	5571	24.86	2096	115200	20.02	820.2	129200	2862
Tc04	23.29	3780	190000	23.93	2686	607000	6621	24.62	1946	169900	23.90	2384	690400	4297
Tc05	24.30	4550	154000	23.43	2585	658000	7243	26.30	2100	122400	23.41	3387	390200	5546
Tc06	24.76	3173	103500	21.91	2862	599700	5978	24.91	2727	113800	23.43	2599	563300	5288
Tc07	23.28	4043	199800	23.94	2363	664100	6604	24.35	2360	150100	24.28	2345	602900	4701
Tc08	24.25	4441	102000	23.03	2192	425000	6578	25.65	1774	112800	21.24	1164	124000	2930
Tc09	23.11	4203	175800	23.74	2429	599000	6859	26.54	1560	110900	24.29	1083	261400	2650
Tc10	23.20	4147	177000	23.68	2638	539000	6998	25.07	2444	110100	20.96	2520	165200	4963
Tc11	23.59	3818	109500	23.68	2201	514800	6088	24.89	2151	118200	24.51	2118	632200	4306
At01	24.92	3023	110400	22.96	3139	695400	6079	25.32	2794	106700	24.17	2978	571400	5748
At02	25.10	2915	125000	23.35	3114	500300	6052	24.29	3475	77010	21.87	2296	576300	5670
At03	23.71	2536	161200	23.97	2975	495000	5545	24.57	2508	159100	24.08	2672	683400	5195
At04	23.78	3819	257000	24.11	1297	799000	5476	23.93	2896	154200	24.14	2201	602000	5217
Asc-8	24.79	2456	120800	20.84	3254	152300	5626	24.98	1825	110900	19.62	1332	219000	3136
Cs01	24.98	2988	109900	22.35	2806	658800	5764	25.27	2667	112900	23.72	2835	575500	5452
Zm01	24.92	3067	102700	22.58	2477	529900	5530	25.36	2502	101400	24.59	2181	491200	4624
Cs02	23.78	4605	75410	22.15	1040	386100	5433	26.00	2655	107000	21.49	1205	193300	3905
Cs03	23.68	3160	223000	24.08	2199	643900	5550	24.83	2195	114500	23.92	2233	483100	4405
Gr01	25.19	2530	137600	23.13	3206	604000	5763	25.18	3178	111500	25.21	2839	612300	6082
Gr02	25.09	3574	91960	22.06	772	310200	4271	24.93	2987	105100	21.34	2606	313200	5518
Mag-2a	23.58	2759	191700	23.93	2773	555900	5649	24.06	4600	96010	22.87	1100	449100	5620
HSP-4	25.87	2339	112300	22.28	1010	160800	3318	26.31	2230	104500	21.80	899	116600	3112
Nat-1	24.86	4197	90360	22.57	893.6	320300	5024	26.05	2164	105900	21.26	271.2	144500	2421
PC & 2:1 PC:PG 2012	23.37	2980	259000	24.09	2070	964000	5210	23.62	2490	339000	24.26	2240	1010000	4810
Q6TV81(25-52)	25.19	2730	117000	22.36	556	346000	3237	25.65	1830	104000				1872
PS - 2 2012	24.17	3290	95700	22.57	1470	514000	4625	24.13	3310	108000	23.09	1780	503000	4926
A4HW34(187-217)	24.45	2500	213000	23.31	1940	485000	4478	25.57	2300	133000	24.03	2450	501000	4699
A5LDU0(184-211)	23.48	2800	268000	24.18	1960	1030000	5015	24.66	2590	134000	24.12	1910	546000	4521
O43312(33-62)	25.31	2810	128000	22.15	2080	512000	4810	24.95	1160	144000				1091
DS 01 2012	24.75	1240	115000	20.49	231	209000	1433	25.77	1730	83100	19.32	309	165000	1982
Nat-1 2012	25.06	2950	108000	21.94	817	242000	3739	26.03	1830	123000	22.18	325	171000	2089
DS 01 (1-12)	23.49	2580	282000	24.18	1800	1060000	4571	23.58	2770	342000	24.22	2280	1010000	5293
Syphaxin	25.43	2260	120000	21.87	194	383000	2416	25.35	3240	101000	22.78	197	485000	3375

P61458(35-60)	23.66	3230	327000	24.15	1180	1090000	4589	25.20	2240	146000	22.37	3000	330000	5254
HSP-4 2012	25.34	2210	94900	20.93	208	214000	2389	26.14	2280	100000	21.49	429	187000	2667
B4FGE3(22-37)	23.52	2970	301000	24.22	2250	1040000	5351	24.20	3010	132000	23.70	2980	492000	5960
A3KLW0(117-136)	23.48	2270	319000	24.20	2110	965000	4362	24.34	3730	165000	23.34	2260	733000	5862
gb|ACU24018.1|(73-101)	23.49	2820	297000	24.20	2210	1010000	5201	23.69	2920	359000	24.24	2360	1010000	5565
gb|AAD22970.1|(120-148)	23.49	2620	294000	24.19	2080	990000	4887	25.23	3830	92000	23.82	1640	393000	5353
Q9XEY7(120-148)	23.49	2570	306000	24.17	2220	909000	5051	25.40	2730	106000	22.23	2060	573000	4791
Penetratin	23.51	2890	295000	24.21	2240	1010000	5358	26.58	2560	116000	24.53	3360	411000	5933
Hyposin (HA-6)	23.48	2590	291000	24.20	2030	1000000	4825	23.65	3110	352000	24.23	2100	1020000	5442
Q8RW88(70-95)	23.49	2670	285000	24.20	2010	1010000	4948	23.82	2980	317000	24.25	1710	974000	4839
B0CZJ3(104-130)	23.5	2670	295000	24.20	2030	1030000	4901	23.74	4050	333000	24.19	1320	1160000	5640
Q8KG25(327-351)	23.49	2600	298000	24.20	2080	1010000	4821	23.58	2360	311000	24.24	2430	894000	5056
Mag-2a 2012	23.32	1870	289000	24.07	3240	703000	5068	23.87	4300	112000	23.71	1040	803000	5289
P94692(929-955)	23.5	2430	304000	24.19	1850	1050000	4381	24.81	2980	122000	21.35	2430	458000	5488
Q7YRI0(9-28)	23.51	2840	286000	24.23	2180	1030000	5279	25.98	1390	152000	22.49	3700	334000	5190
Pseudin-B	24.57	2320	90700	23.04	2330	277000	4536	24.96	2200	106000	20.92	1400	176000	3575

Legend:

Tm = transition temperature, given in °C

Broad = broad component of the transition

Sharp = sharp component of the transition

Heat = total area under de curve

Pc = DMPC LUVs

Pcpg = 2:1 DMPC:DMPG LUVs

ΔH = transition enthalpy, given in kcal.mol^{-1}

ΔHv = van´t Hoff transition enthalpy, given in kcal.mol^{-1}

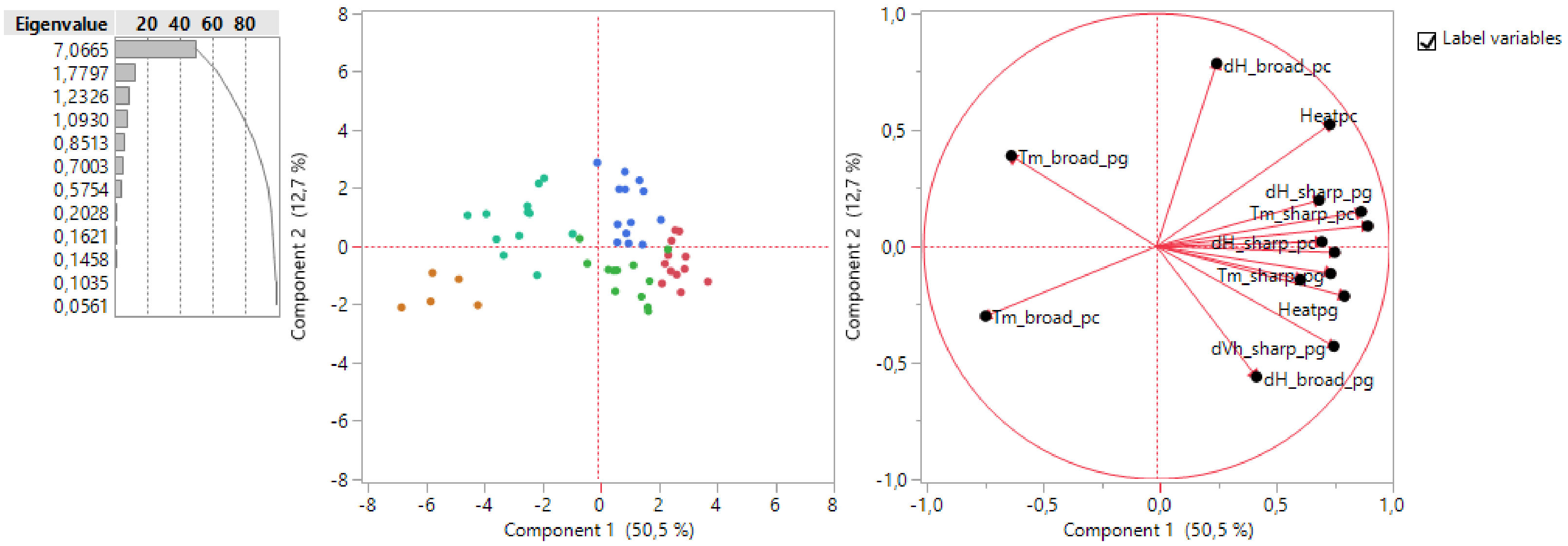

Figure S2.1. Summary plot of the PCA2. Cluster 1, 2, 3, 4 and 5 peptides are represented as red, blue, green, light green and orange dots, respectively.

Legend:

Tm = transition temperature, given in °C
Broad = broad component of the transition
Sharp = sharp component of the transition
Heat = total area under de curve
Pc = DMPC LUVs
Pcpg = 2:1 DMPC:DMPG LUVs
ΔH = transition enthalpy, given in kcal.mol^{-1}
ΔHv = van´t Hoff transition enthalpy, given in kcal.mol^{-1}

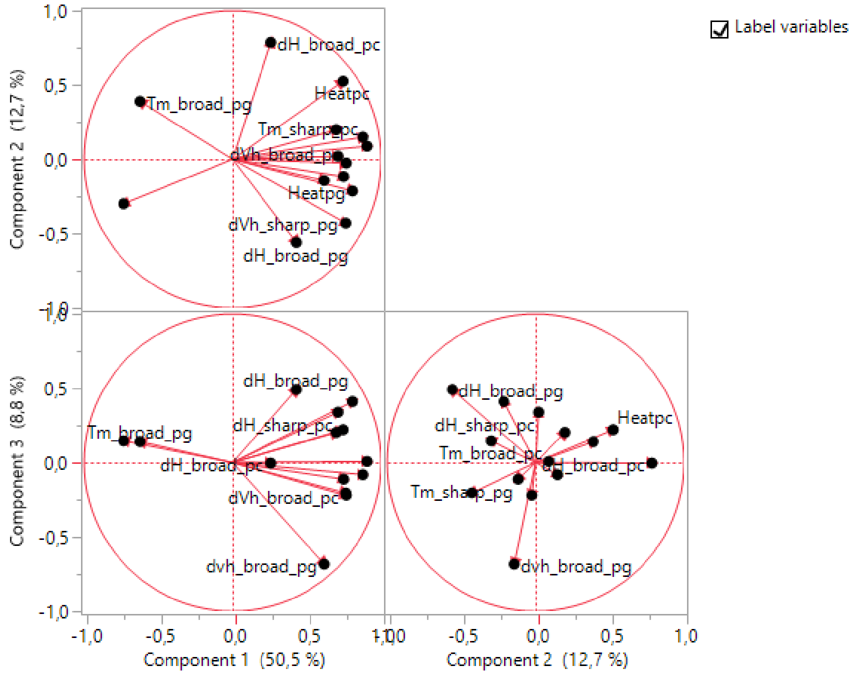

	Prin1	Prin2	Prin3	Prin4	Prin5
Tm_broad_pc	-0,73414	-0,29925	0,14631	0,48824	-0,02923
dH_broad_pc	0,25746	0,78558	-0,00393	-0,04904	-0,44757
dVh_broad_pc	0,76468	-0,02519	-0,22009	-0,35356	0,42116
Tm_sharp_pc	0,87838	0,14937	-0,08058	-0,21863	0,17444
dH_sharp_pc	0,70926	0,02045	0,33892	0,20280	0,04109
dVh_sharp_pc	0,90639	0,08800	0,00780	0,01455	0,20368
Heatpc	0,74338	0,52366	0,22122	0,05127	-0,20272
Tm_broad_pg	-0,62408	0,38930	0,14113	0,21605	0,49896
dH_broad_pg	0,42829	-0,56005	0,49190	-0,36632	-0,17937
dvh_broad_pg	0,61704	-0,14278	-0,68341	0,13305	-0,10379
Tm_sharp_pg	0,74686	-0,11639	-0,11093	0,39086	0,02375
dH_sharp_pg	0,69771	0,19867	0,20294	0,43701	0,15753
dVh_sharp_pg	0,76108	-0,42840	-0,20371	0,30413	-0,19827
Heatpg	0,80640	-0,21348	0,41045	0,02438	0,03839

Figure S2.2. Loading plot of the first three principal components of the PCA1 analysis and loading matrix of the first five principal components.

Legend:

Tm = transition temperature, given in °C

Broad = broad component of the transition

Sharp = sharp component of the transition

Heat = total area under de curve

Pc = DMPC LUVs

Pcpg = 2:1 DMPC:DMPG LUVs

ΔH = transition enthalpy, given in $kcal.mol^{-1}$

ΔHv = van´t Hoff transition enthalpy, given in $kcal.mol^{-1}$

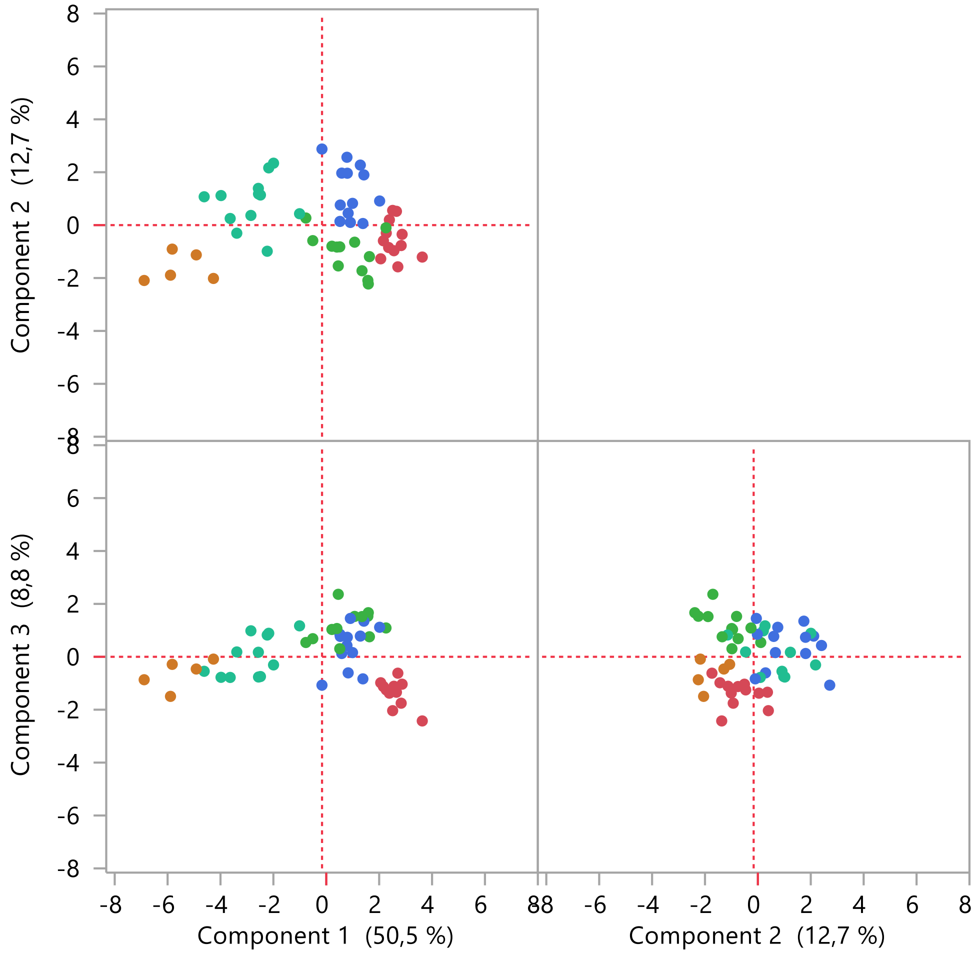

Figure S2.3. Score plot of the first three principal components of the PCA1. Cluster 1, 2 and 3 peptides are represented as red, green and blue dots, respectively.

Number	Eigenvalue	Percent		Cum Percent
1	7,0665	50,475		50,475
2	1,7797	12,712		63,187
3	1,2326	8,804		71,992
4	1,0930	7,807		79,799
5	0,8513	6,081		85,879
6	0,7003	5,002		90,882
7	0,5754	4,110		94,991
8	0,2028	1,449		96,440
9	0,1621	1,158		97,597
10	0,1458	1,042		98,639
11	0,1035	0,739		99,378
12	0,0561	0,401		99,779
13	0,0205	0,146		99,926
14	0,0104	0,074		100,000

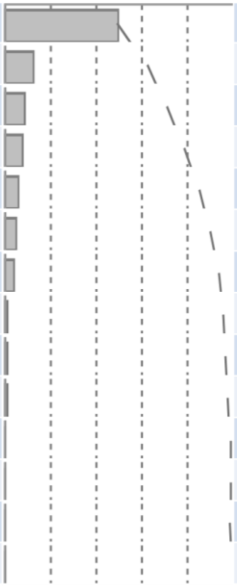

Figure S2.4. Eigenvalues of the PCA2 analysis.

Supplementary material 03

Table S3. Peptides Mean Residue Ellipcity (MRE) in buffer and titrated with LUVs and their percent of a perfect helical segment according to the methodology of Chen *et al.*1974.

Peptide	$[\theta]_{222}$ nm Buffer	[θ]222 nm 0.01 peptide/DMPC (mol/mol)	[θ]222 nm 0.01 peptide/(2:1 DMPC/DMPG) (mol/mol)	Maximum theoretical helicity	% Helicity (Buffer))	% Helicity 0.01 peptide/DMPC (mol/mol)	% Helicity 0.01 peptide/(2:1 DMPC/DMPG) (mol/mol)
Tc01	-1665	-1498	-21476	-33155	**5.0**	**4.5**	**64.8**
Tc02	-20797	-22588	-26496	-34424	**60.4**	**65.6**	**77.0**
Tc03	-13991	-12765	-17587	-34666	**40.4**	**36.8**	**50.7**
Tc04	-4993	-5509	-12653	-34886	**14.3**	**15.8**	**36.3**
Tc05	-2866	-12024	-23686	-34424	**8.3**	**34.9**	**68.8**
Tc06	-10054	-10757	-11930	-34157	**29.4**	**31.5**	**34.9**
Tc07	-	-	-	-	**-**	**-**	**-**
Tc08	-3129	-14082	-21667	-34886	**9.0**	**40.4**	**62.1**
Tc09	-4230	-6659	259	-34424	**12.3**	**19.3**	**-0.8**
Tc10	-6951	-1734	-16933	-34157	**20.3**	**5.1**	**49.6**
Tc11	-15362	-19119	-19018	-33529	**45.8**	**57.0**	**56.7**
Asc-8	-917	-25343	-30237	-34157	**2.7**	**74.2**	**88.5**
DS01 2016	-8350	-21369	-26052	-35999	**23.2**	**59.4**	**72.4**
At01	-11053	-9021	-9696	-34157	**32.4**	**26.4**	**28.4**
At02	-4455	-10919	-26743	-33529	**13.3**	**32.6**	**79.8**
At03	421	390	-10412	-33860	**-1.2**	**-1.2**	**30.8**
At04	-	-	-	-	**-**	**-**	**-**
Cs01	-8972	-12918	-9407	-34157	**26.3**	**37.8**	**27.5**
Zm01	-10590	-14424	-22372	-34157	**31.0**	**42.2**	**65.5**
Cs02	-1439	-17868	-17910	-33860	**4.3**	**52.8**	**52.9**
Cs03	-	-	-	-	**-**	**-**	**-**
Gr01	-307	-19129	-38128	-33155	**0.9**	**57.7**	**115.0**
Gr02	-19380	-17238	-21933	-34157	**56.7**	**50.5**	**64.2**
HSP-4 2016	-4412	-34784	-34224	-35439	**12.4**	**98.1**	**96.6**

Mag-2a 2016	-2614	-621	-16691	-35086	**7.5**	**1.8**	**47.6**
Nat-1 2016	-4639	-17321	-28722	-36116	**12.8**	**48.0**	**79.5**
PS-2 2016	-3579	-14514	-20402	-34157	**10.5**	**42.5**	**59.7**
A5LDU0(184-211)	-	-	-	-	**-**	**-**	**-**
HSP-4 2012	-4148	-33266	-31838	-35270	**11.8**	**94.3**	**90.3**
Q8KG25(327-351)	-6669	-5287	-6002	-35439	**18.8**	**14.9**	**16.9**
O43312(33-62)	-4308	-15500	-20372	-36116	**11.9**	**42.9**	**56.4**
Q6TV81(25-52)	-17113	-27515	-35902	-35875	**47.7**	**76.7**	**100.1**
A3KLW0(117-136)	-3442	-3087	-3008	-34424	**10.0**	**9.0**	**8.7**
Syphaxin	-3425	-24171	-27196	-35439	**9.7**	**68.2**	**76.7**
PS-2 2012	-1915	-10965	-11416	-34157	**5.6**	**32.1**	**33.4**
A4HW34(187-217)	-2190	-6329	-11588	-36225	**6.0**	**17.5**	**32.0**
Mag-2a 2012	-801	-1173	-13502	-35086	**2.3**	**3.3**	**38.5**
Q7YRI0(9-28)	-4615	-4134	-15064	-34424	**13.4**	**12.0**	**43.8**
B4FGE3(22-37)	-1506	-1051	-2047	-33155	**4.5**	**3.2**	**6.2**
P61458(35-60)	-2711	-2184	-8352	-35596	**7.6**	**6.1**	**23.5**
DS 01 (1-12)	-2137	-1312	-3767	-31040	**6.9**	**4.2**	**12.1**
Penetratin	-2593	-1655	-9813	-33155	**7.8**	**5.0**	**29.6**
P94692(929-955)	-2066	-2899	-12242	-35740	**5.8**	**8.1**	**34.3**
gb|ACU24018.1|(73-101)	-2129	-1898	-2273	-35875	**5.9**	**5.3**	**6.3**
gb|AAD22970.1|(120-148)	-	-	-	-	**-**	**-**	**-**
Nat-1 2012	-5170	-23159	-27271	-36116	**14.3**	**64.1**	**75.5**
B0CZJ3(104-130)	-7158	-5729	-6900	-35740	**20.0**	**16.0**	**19.3**
Hyposin HA-6	-5194	-3848	-4989	-32249	**16.1**	**11.9**	**15.5**
Q9XEY7(120-148)	-2931	-3183	-12158	-36000	**8.1**	**8.8**	**33.8**
Q8RW88(70-95)	-6062	-4406	-5317	-35596	**17.0**	**12.4**	**14.9**
DS 01 2012	-7302	-31124	-30034	-36000	**20.3**	**86.5**	**83.4**
Pseudin B	-5203	-25330	-24121	-35086	**14.8**	**72.2**	**68.7**

Supplementary material 04

Table S4.1. Peptide names, number of residues, physicochemical properties and corresponding cluster

Peptide name	Number of residues	Net charge	Mol. Mass (Da)	Aggregation (Na4vSS)	GOR IV Hel	GOR IV Ext	GOR IV Rand	Avg. Hydrophobicity (TM scale)	Hyd. Moment (TM scale)	Cluster
PS-2	19	3	2115,1628	36,6547	0	36,84	63,16	0,43579	0,896651	Cluster 3
DS01	29	3	2792,5864	-11,0462	62,07	10,34	27,59	-0,25172	0,385957	Cluster 5
Tc02	20	5	2462,5457	50,385	40	30	30	0,0675	1,07247	Cluster 4
Tc01	16	2	1836,0939	1,8863	0	50	50	-0,24875	0,860646	Cluster 3
Tc03	21	2	2599,5776	19,8895	14,29	38,1	47,62	-0,28714	0,436778	Cluster 4
Tc04	22	1	2480,407	6,542	36,36	18,18	45,45	-0,37682	0,847849	Cluster 1
Tc05	20	3	2290,34	15,974	0	40	60	-0,11	1,02677	Cluster 2
Tc06	19	3	2273,169	32,515	0	47,37	52,63	0,208947	0,583667	Cluster 3
Tc07	20	5	2424,349	17,013	0	45	55	-0,025	0,622236	Cluster 1
Tc08	22	5	2460,449	14,144	36,36	13,64	50	-0,18818	0,795542	Cluster 4
Tc09	20	3	2347,415	25,389	35	25	40	-0,292	0,487917	Cluster 2
Tc10	19	3	2305,388	35,322	47,37	21,05	31,58	-0,11947	0,720198	Cluster 2
Tc11	17	1	1936,229	20,994	29,41	17,65	52,94	0,011177	0,7576	Cluster 2
At01	19	3	2260,1741	38,5305	0	47,37	52,63	0,277368	0,585786	Cluster 3
At02	16	4	1731,1339	37,6	25	31,25	43,75	0,098125	0,889916	Cluster 3
At03	18	5	1994,1781	5,9467	33,33	16,67	50	-0,38778	0,958448	Cluster 1
At04	20	3	2361,32	38,506	0	40	60	0,1715	0,982197	Cluster 1
Asc-8	19	3	2017,229	14,325	42,11	21,05	36,84	-0,13211	0,881728	Cluster 4
Cs01	19	3	2301,17	30,8884	0	52,63	47,37	0,255789	0,537373	Cluster 3
Zm01	19	4	2300,27	39,4516	0	57,89	42,11	0,171053	0,589726	Cluster 3
Cs02	18	3	2175,11	11,7756	0	50	50	0,192778	0,865522	Cluster 4
Cs03	18	3	2119,124	24,87	0	50	50	0,023684	0,947115	Cluster 2
Gr01	16	5	1930,2236	36,7438	0	50	50	-0,05	1,11154	Cluster 3
Gr02	19	3	2261,3035	14,3053	47,37	10,53	42,11	-0,14947	0,943109	Cluster 4
Mag-2a	23	4	2465,3252	8,0043	17,39	43,48	39,13	-0,27391	0,872665	Cluster 3
HSP-4	25	2	2416,3799	-9,8784	60	8	32	-0,2376	0,700737	Cluster 5
Nat-1	30	3	3178,7898	-8,552	53,33	6,67	40	-0,55267	0,270437	Cluster 4

Q6TV81(25-52)	28	4	2983,686	-2,3571	64,29	14,29	21,43	-0,38571	0,708544	Cluster 4
A4HW34(187-217)	31	4	3402,7769	-2,0058	61,29	9,68	29,03	-0,19387	0,611147	Cluster 3
A5LDU0(184-211)	28	6	3237,8208	16,6436	0	50	50	-0,31893	0,383199	Cluster 2
O43312(33-62)	30	3	3205,7971	2,8547	60	6,67	33,33	-0,14367	0,59434	Cluster 4
DS 01 (1-12)	12	2	1373,7667	-27,9983	0	41,67	58,33	-0,91917	0,784731	Cluster 1
Syphaxin	25	3	2543,5164	4,06	44	32	24	-0,2824	0,377021	Cluster 5
P61458(35-60)	26	4	3175,6536	0,1792	0	42,31	57,69	-0,36231	0,569537	Cluster 2
B4FGE3(22-37)	16	4	1800,0887	12,845	0	37,5	62,5	-0,24563	0,623714	Cluster 2
A3KLW0(117-136)	20	3	2402,416	5,07	30	20	50	-0,432	0,457721	Cluster 3
gb|ACU24018.1|(73-101)	28	0	3164,5735	-56,7921	17,86	21,43	60,71	-1,23964	0,150136	Cluster 1
gb|AAD22970.1|(120-148)	28	4	3181,5503	-23,6707	39,29	25	35,71	-0,4425	0,267436	Cluster 2
Q9XEY7(120-148)	29	4	3125,7705	-3,8235	13,79	17,24	68,97	-0,40069	0,583536	Cluster 2
Penetratin	16	7	2245,2893	-18,6338	0	75	25	-0,225	0,56454	Cluster 2
Hyposin (HA-6)	14	4	1519,0087	10,15	0	64,29	35,71	-0,32957	0,0866461	Cluster 1
Q8RW88(70-95)	26	2	2630,335	-13,5992	23,08	38,46	38,46	-0,32269	0,53686	Cluster 1
B0CZJ3(104-130)	27	4	2740,4885	-13,4763	40,74	7,41	51,85	-0,23148	0,598763	Cluster 1
Q8KG25(327-351)	25	2	2685,5176	-0,7488	20	24	56	-0,3708	0,523225	Cluster 1
P94692(929-955)	27	4	2930,6987	-7,7711	33,33	25,93	40,74	-0,61037	0,576262	Cluster 2
Q7YRI0(9-28)	20	6	2348,4602	-7,22	35	35	30	-0,455	0,64189	Cluster 2
Pseudin-B	23	4	2537,4807	0,2435	56,52	8,7	34,78	-0,2824	1,01637	Cluster 4

Oneway Analysis of N. res

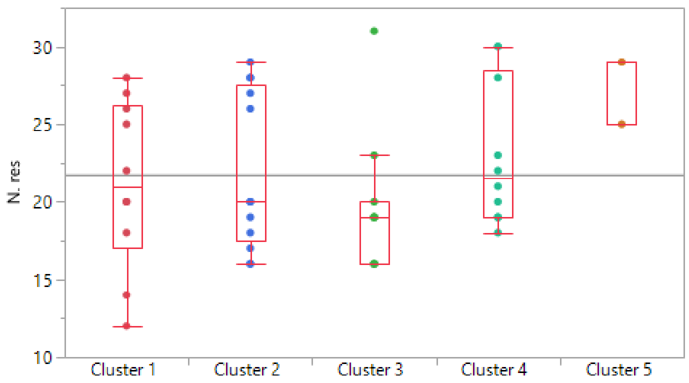

Quantiles

Level	Minimum	10%	25%	Median	75%	90%	Maximum
Cluster 1	12	12,2	17	21	26,25	27,9	28
Cluster 2	16	16	17,5	20	27,5	28,6	29
Cluster 3	16	16	16	19	20	29,4	31
Cluster 4	18	18,1	19	21,5	28,5	30	30
Cluster 5	25	25	25	25	29	29	29

Nonparametric Comparisons For All Pairs Using Steel-Dwass Method

q*	Alpha
2,72777	0,05

Level	- Level	Score Mean Difference	Std Err Dif	Z	p-Value	Hodges-Lehmann	Lower CL	Upper CL
Cluster 5	Cluster 3	5,51515	2,648833	2,08211	0,2278	6,00000	.	.
Cluster 4	Cluster 3	4,67727	2,655055	1,76165	0,3962	3,00000	-1,00000	11,00000
Cluster 5	Cluster 2	3,48718	3,031459	1,15033	0,7796	5,00000	.	.
Cluster 5	Cluster 1	3,25000	2,545966	1,27653	0,7057	5,00000	.	.
Cluster 5	Cluster 4	2,81667	2,553048	1,10326	0,8049	4,50000	.	.
Cluster 4	Cluster 2	2,56538	2,837961	0,90395	0,8955	2,00000	-7,00000	10,00000
Cluster 4	Cluster 1	1,60000	2,636784	0,60680	0,9741	1,50000	-7,00000	10,00000
Cluster 2	Cluster 1	0,53077	2,832997	0,18735	0,9997	0,00000	-8,00000	8,00000
Cluster 3	Cluster 1	-2,57727	2,686329	-0,95940	0,8733	-2,00000	-9,00000	6,00000
Cluster 3	Cluster 2	-3,02098	2,854962	-1,05815	0,8279	-1,00000	-9,00000	3,00000

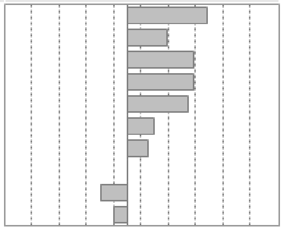

Oneway Analysis of Net charge

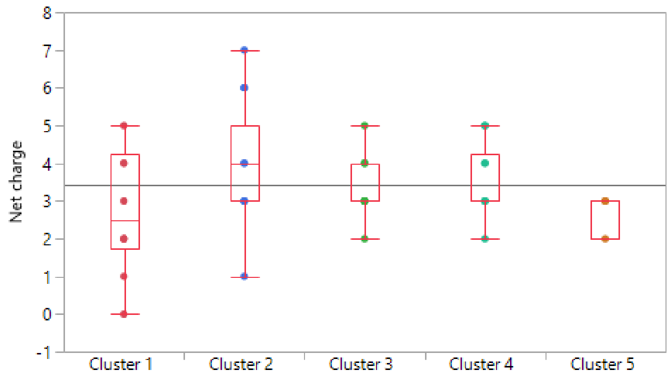

Quantiles

Level	Minimum	10%	25%	Median	75%	90%	Maximum
Cluster 1	0	0,1	1,75	2,5	4,25	5	5
Cluster 2	1	1,8	3	4	5	6,6	7
Cluster 3	2	2,2	3	3	4	4,8	5
Cluster 4	2	2,1	3	3	4,25	5	5
Cluster 5	2	2	2	3	3	3	3

Level	- Level	Score Mean Difference	Std Err Dif	Z	p-Value	Hodges-Lehmann	Lower CL	Upper CL
Cluster 2	Cluster 1	4,06923	2,793693	1,45658	0,5909	1,00000	-1,00000	4,000000
Cluster 4	Cluster 1	2,50000	2,580290	0,96888	0,8692	1,00000	-2,00000	3,000000
Cluster 3	Cluster 1	2,38636	2,636113	0,90526	0,8950	1,00000	-1,00000	3,000000
Cluster 4	Cluster 3	0,00000	2,524201	0,00000	1,0000	0,00000	-1,00000	1,000000
Cluster 5	Cluster 1	0,00000	2,506658	0,00000	1,0000	0,00000	.	.
Cluster 4	Cluster 2	-2,38846	2,724468	-0,87667	0,9056	0,00000	-3,00000	1,000000
Cluster 3	Cluster 2	-2,51748	2,740860	-0,91850	0,8899	0,00000	-2,00000	1,000000
Cluster 5	Cluster 4	-3,03333	2,346688	-1,29260	0,6958	-1,00000	.	.
Cluster 5	Cluster 3	-3,60606	2,516148	-1,43317	0,6061	-1,00000	.	.
Cluster 5	Cluster 2	-4,92308	2,921187	-1,68530	0,4430	-1,00000	.	.

Oneway Analysis of Mol mass

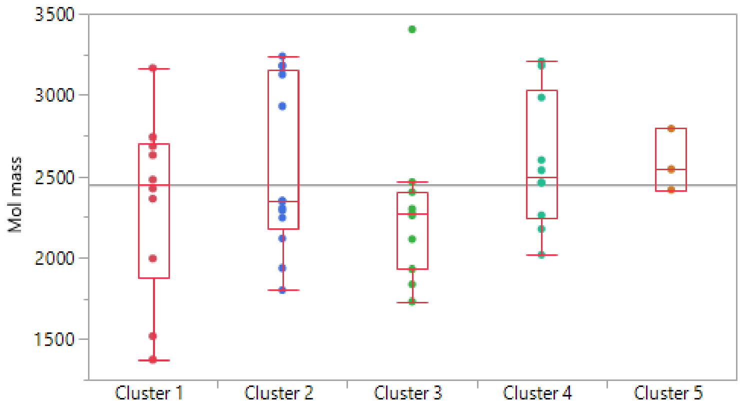

Quantiles

Level	Minimum	10%	25%	Median	75%	90%	Maximum
Cluster 1	1373,767	1388,291	1875,386	2452,378	2699,26	3122,165	3164,574
Cluster 2	1800,089	1854,545	2182,207	2347,415	3150,712	3215,313	3237,821
Cluster 3	1731,134	1752,126	1930,224	2273,169	2402,416	3215,287	3402,777
Cluster 4	2017,229	2033,017	2239,755	2500,013	3032,462	3203,096	3205,797
Cluster 5	2416,38	2416,38	2416,38	2543,516	2792,586	2792,586	2792,586

Level	- Level	Score Mean Difference	Std Err Dif	Z	p-Value	Hodges-Lehmann	Lower CL	Upper CL
Cluster 5	Cluster 3	5,09091	2,724746	1,86840	0,3345	327,261	.	.
Cluster 4	Cluster 3	4,67727	2,711088	1,72524	0,4183	292,251	-242,945	905,621
Cluster 4	Cluster 1	1,90000	2,645751	0,71813	0,9524	147,200	-479,185	1080,569
Cluster 5	Cluster 2	1,64103	3,049450	0,53814	0,9834	171,091	.	.
Cluster 5	Cluster 1	1,51667	2,563635	0,59161	0,9764	113,118	.	.
Cluster 4	Cluster 2	1,32692	2,852799	0,46513	0,9904	80,513	-713,108	799,489
Cluster 2	Cluster 1	0,79615	2,852799	0,27908	0,9987	217,696	-544,178	876,501
Cluster 5	Cluster 4	0,21667	2,563635	0,08452	1,0000	43,503	.	.
Cluster 3	Cluster 1	-2,76818	2,711088	-1,02106	0,8458	-179,687	-794,241	782,161
Cluster 3	Cluster 2	-3,35664	2,896827	-1,15873	0,7749	-188,900	-936,651	315,074

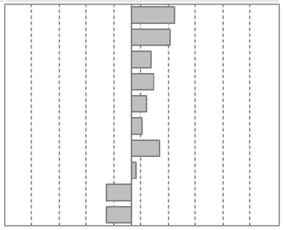

Oneway Analysis of Agg

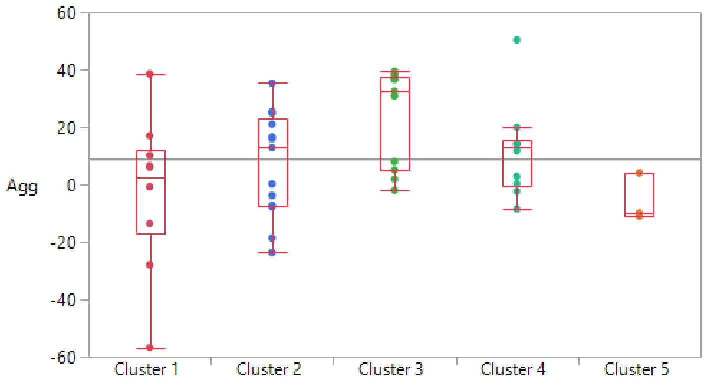

Quantiles

Level	Minimum	10%	25%	Median	75%	90%	Maximum
Cluster 1	-56,7921	-53,9127	-17,199	2,59895	11,86575	36,3567	38,506
Cluster 2	-23,6707	-21,6559	-7,49555	12,845	22,932	31,3488	35,322
Cluster 3	-2,0058	-1,22738	5,07	32,515	37,6	39,26738	39,4516
Cluster 4	-8,552	-7,93251	-0,40665	12,9598	15,71613	47,33545	50,385
Cluster 5	-11,0462	-11,0462	-11,0462	-9,8784	4,06	4,06	4,06

Level	- Level	Score Mean Difference	Std Err Dif	Z	p-Value	Hodges-Lehmann	Lower CL	Upper CL
Cluster 3	Cluster 2	6,71329	2,896827	2,31746	0,1392	15,8714	-10,9587	43,96380
Cluster 3	Cluster 1	5,82273	2,711088	2,14775	0,2000	27,0219	-5,0800	60,51330
Cluster 4	Cluster 1	3,70000	2,645751	1,39847	0,6286	11,1807	-16,7695	51,13380
Cluster 2	Cluster 1	2,74231	2,852799	0,96127	0,8725	9,5424	-21,8624	40,84330
Cluster 4	Cluster 2	0,44231	2,852799	0,15504	0,9999	1,4634	-21,0167	29,39100
Cluster 5	Cluster 1	-0,65000	2,563635	-0,25355	0,9991	-4,2860	.	.
Cluster 5	Cluster 2	-3,69231	3,049450	-1,21081	0,7451	-11,9140	.	.
Cluster 4	Cluster 3	-3,91364	2,711088	-1,44357	0,5993	-17,9503	-37,3565	13,64120
Cluster 5	Cluster 4	-4,55000	2,563635	-1,77482	0,3883	-13,3170	.	.
Cluster 5	Cluster 3	-5,93939	2,724746	-2,17980	0,1873	-33,5400	.	.

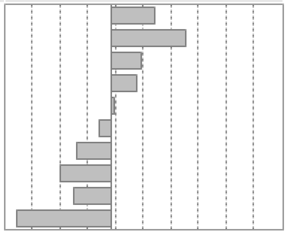

Oneway Analysis of Gor Hel

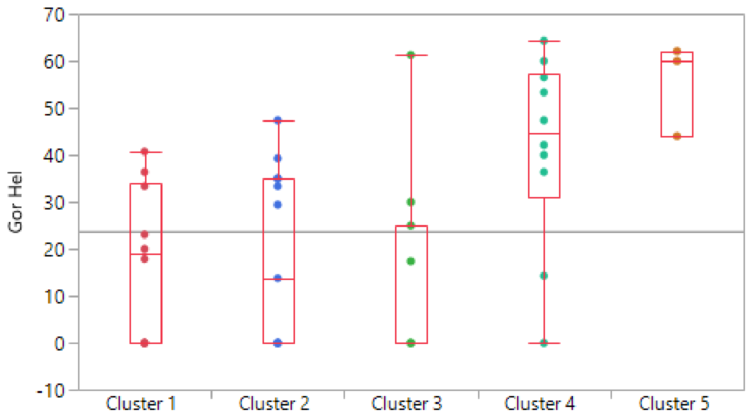

Quantiles

Level	Minimum	10%	25%	Median	75%	90%	Maximum
Cluster 1	0	0	0	18,93	34,0875	40,302	40,74
Cluster 2	0	0	0	13,79	35	44,138	47,37
Cluster 3	0	0	0	0	25	55,032	61,29
Cluster 4	0	1,429	30,8425	44,74	57,39	63,861	64,29
Cluster 5	44	44	44	60	62,07	62,07	62,07

Level	- Level	Score Mean Difference	Std Err Dif	Z	p-Value	Hodges-Lehmann	Lower CL	Upper CL
Cluster 4	Cluster 2	7,78462	2,811627	2,76872	0,0446*	24,46000	0,0000	53,33000
Cluster 5	Cluster 2	7,38462	2,967632	2,48839	0,0932	44,00000	.	.
Cluster 4	Cluster 3	6,87273	2,636113	2,60714	0,0690	36,36000	-1,2900	56,52000
Cluster 4	Cluster 1	6,60000	2,624781	2,51450	0,0874	24,27000	-3,5700	53,33000
Cluster 5	Cluster 1	6,28333	2,528175	2,48532	0,0939	41,03500	.	.
Cluster 5	Cluster 3	5,93939	2,551566	2,32774	0,1361	44,00000	.	.
Cluster 5	Cluster 4	3,03333	2,560111	1,18484	0,7601	8,19000	.	.
Cluster 2	Cluster 1	0,00000	2,732574	0,00000	1,0000	0,00000	-23,0800	33,33000
Cluster 3	Cluster 1	-2,38636	2,509980	-0,95075	0,8769	0,00000	-33,3300	25,00000
Cluster 3	Cluster 2	-2,51748	2,657047	-0,94747	0,8782	0,00000	-35,0000	25,00000

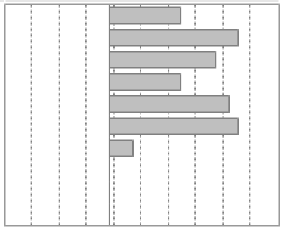

Oneway Analysis of Gor Ex

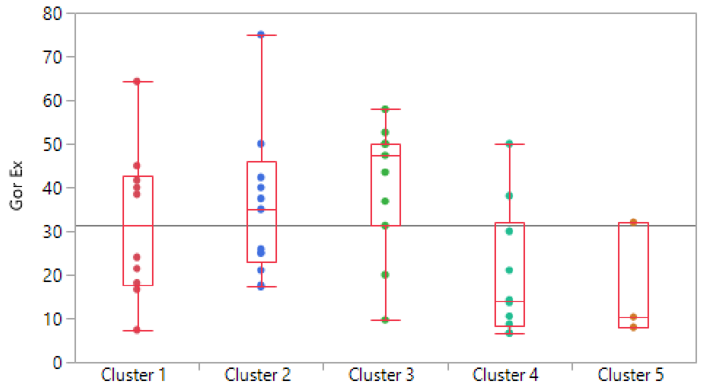

Quantiles

Level	Minimum	10%	25%	Median	75%	90%	Maximum
Cluster 1	7,41	8,336	17,8025	31,23	42,5025	62,361	64,29
Cluster 2	17,24	17,404	23,025	35	46,155	65	75
Cluster 3	9,68	11,744	31,25	47,37	50	56,838	57,89
Cluster 4	6,67	6,67	8,1925	13,965	32,025	48,81	50
Cluster 5	8	8	8	10,34	32	32	32

Level	- Level	Score Mean Difference	Std Err Dif	Z	p-Value	Hodges-Lehmann	Lower CL	Upper CL
Cluster 3	Cluster 1	3,91364	2,709327	1,44450	0,5987	9,9100	-16,9200	33,33000
Cluster 3	Cluster 2	3,18881	2,889260	1,10368	0,8047	7,8900	-17,5000	26,32000
Cluster 2	Cluster 1	1,59231	2,850684	0,55857	0,9809	3,2200	-20,6200	28,57000
Cluster 5	Cluster 4	-0,65000	2,560111	-0,25390	0,9991	-1,6150	.	.
Cluster 5	Cluster 1	-3,25000	2,563635	-1,26773	0,7111	-12,0450	.	.
Cluster 4	Cluster 1	-4,70000	2,644756	-1,77710	0,3870	-10,6300	-34,4700	13,33000
Cluster 5	Cluster 3	-5,09091	2,718751	-1,87252	0,3322	-25,8900	.	.
Cluster 5	Cluster 2	-5,33333	3,044962	-1,75153	0,4023	-17,0000	.	.
Cluster 4	Cluster 2	-6,54615	2,847861	-2,29862	0,1452	-14,4250	-35,7100	5,00000
Cluster 4	Cluster 3	-6,77727	2,705802	-2,50472	0,0895	-23,8900	-41,3000	2,63000

Oneway Analysis of Gor Rand

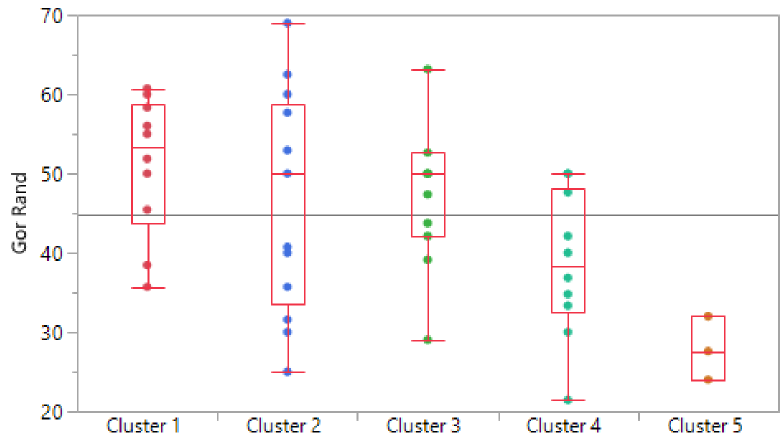

Quantiles

Level	Minimum	10%	25%	Median	75%	90%	Maximum
Cluster 1	35,71	35,985	43,7025	53,425	58,7475	60,639	60,71
Cluster 2	25	27	33,645	50	58,845	66,382	68,97
Cluster 3	29,03	31,05	42,11	50	52,63	61,054	63,16
Cluster 4	21,43	22,287	32,4975	38,42	48,215	50	50
Cluster 5	24	24	24	27,59	32	32	32

Level	- Level	Score Mean Difference	Std Err Dif	Z	p-Value	Hodges-Lehmann	Lower CL	Upper CL
Cluster 3	Cluster 2	0,00000	2,883572	0,00000	1,0000	0,0000	-16,2500	18,42000
Cluster 2	Cluster 1	-2,21154	2,848567	-0,77637	0,9375	-4,8550	-22,6200	12,50000
Cluster 3	Cluster 1	-2,86364	2,701389	-1,06006	0,8270	-5,3500	-16,4200	11,54000
Cluster 4	Cluster 2	-3,98077	2,844329	-1,39955	0,6280	-7,8900	-25,6600	10,00000
Cluster 5	Cluster 4	-4,55000	2,560111	-1,77727	0,3869	-10,4450	.	.
Cluster 4	Cluster 3	-5,34545	2,691654	-1,98594	0,2728	-8,8750	-22,3200	5,51000
Cluster 5	Cluster 2	-6,15385	3,047207	-2,01950	0,2565	-18,0000	.	.
Cluster 5	Cluster 1	-6,28333	2,563635	-2,45095	0,1021	-25,1300	.	.
Cluster 5	Cluster 3	-6,36364	2,709734	-2,34844	0,1298	-19,7800	.	.
Cluster 4	Cluster 1	-6,90000	2,641770	-2,61189	0,0682	-12,6350	-26,0000	1,54000

Oneway Analysis of Avg Hyd

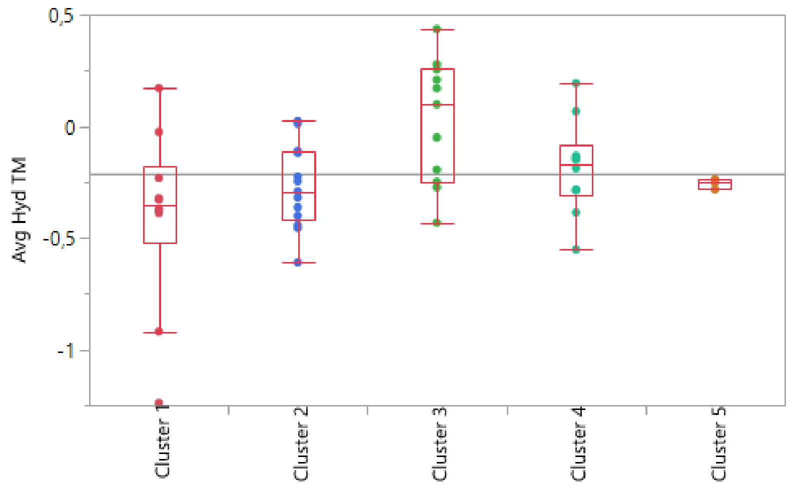

Quantiles

Level	Minimum	10%	25%	Median	75%	90%	Maximum
Cluster 1	-1,23964	-1,20759	-0,52063	-0,35018	-0,17986	0,15185	0,1715
Cluster 2	-0,61037	-0,54822	-0,4216	-0,292	-0,11474	0,018681	0,023684
Cluster 3	-0,432	-0,40038	-0,24875	0,098125	0,255789	0,404106	0,43579
Cluster 4	-0,55267	-0,53597	-0,31179	-0,16883	-0,0822	0,18025	0,192778
Cluster 5	-0,2824	-0,2824	-0,2824	-0,25172	-0,2376	-0,2376	-0,2376

Level	- Level	Score Mean Difference	Std Err Dif	Z	p-Value	Hodges-Lehmann	Lower CL	Upper CL
Cluster 3	Cluster 2	7,21678	2,896827	2,49127	0,0925	0,312310	-0,061177	0,6396780
Cluster 3	Cluster 1	6,58636	2,711088	2,42942	0,1075	0,434061	-0,055180	0,9908900
Cluster 4	Cluster 1	3,90000	2,645751	1,47406	0,5794	0,176120	-0,257400	0,7755000
Cluster 4	Cluster 2	2,74231	2,852799	0,96127	0,8725	0,087110	-0,177140	0,3595000
Cluster 5	Cluster 1	2,38333	2,563635	0,92967	0,8855	0,090185	.	.
Cluster 2	Cluster 1	1,85769	2,852799	0,65118	0,9665	0,054880	-0,280800	0,6271700
Cluster 5	Cluster 2	0,82051	3,049450	0,26907	0,9989	0,036530	.	.
Cluster 5	Cluster 4	-1,30000	2,560111	-0,50779	0,9866	-0,091030	.	.
Cluster 5	Cluster 3	-4,24242	2,724746	-1,55700	0,5252	-0,349845	.	.
Cluster 4	Cluster 3	-4,48636	2,711088	-1,65482	0,4622	-0,234770	-0,579460	0,1448600

Oneway Analysis of Hyd Mom

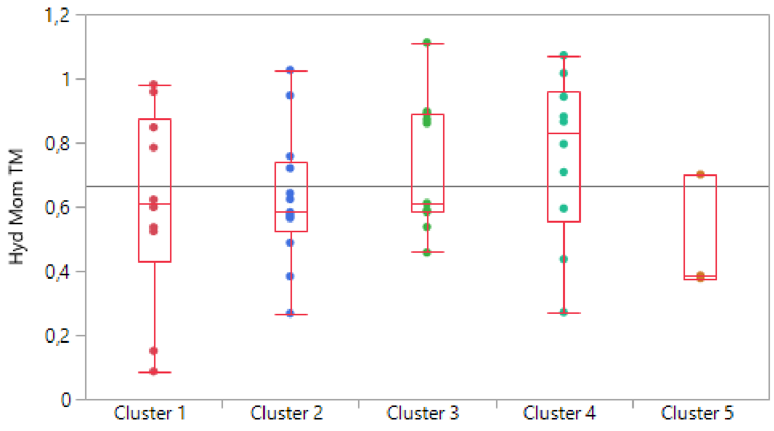

Quantiles

Level	Minimum	10%	25%	Median	75%	90%	Maximum
Cluster 1	0,086646	0,092995	0,429953	0,6105	0,875499	0,979822	0,982197
Cluster 2	0,267436	0,313741	0,526229	0,583536	0,738899	0,994908	1,02677
Cluster 3	0,457721	0,473651	0,583667	0,611147	0,889916	1,068562	1,11154
Cluster 4	0,270437	0,287071	0,55495	0,830532	0,961424	1,06686	1,07247
Cluster 5	0,377021	0,377021	0,377021	0,385957	0,700737	0,700737	0,700737

Level	- Level	Score Mean Difference	Std Err Dif	Z	p-Value	Hodges-Lehmann	Lower CL	Upper CL
Cluster 4	Cluster 2	3,98077	2,852799	1,39539	0,6306	0,165436	-0,217480	0,4487560
Cluster 3	Cluster 2	3,02098	2,896827	1,04286	0,8354	0,084770	-0,154105	0,3539400
Cluster 4	Cluster 1	2,90000	2,645751	1,09610	0,8087	0,139340	-0,273653	0,6218979
Cluster 3	Cluster 1	1,81364	2,711088	0,66897	0,9631	0,056654	-0,310476	0,5030799
Cluster 4	Cluster 3	1,24091	2,711088	0,45772	0,9910	0,049825	-0,315998	0,4057360
Cluster 2	Cluster 1	-0,08846	2,852799	-0,03101	1,0000	-0,013280	-0,358483	0,4280070
Cluster 5	Cluster 1	-1,95000	2,563635	-0,76064	0,9418	-0,155371	.	.
Cluster 5	Cluster 2	-2,87179	3,049450	-0,94174	0,8806	-0,187519	.	.
Cluster 5	Cluster 4	-4,11667	2,563635	-1,60579	0,4936	-0,319110	.	.
Cluster 5	Cluster 3	-4,24242	2,724746	-1,55700	0,5252	-0,203769	.	.

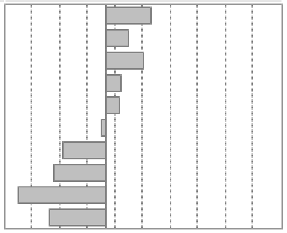

Supplement: Supplementary file 1 — Supplementary material [file 41598_2018_19566_MOESM1_ESM.pdf]
